# 3D virtual biopsy of in vivo pH and metabolism using PRESS and semi‐LASER MRS of hyperpolarized ^13^C nuclei

**DOI:** 10.1002/mrm.30544

**Published:** 2025-05-01

**Authors:** Wolfgang Gottwald, Luca Nagel, Martin Grashei, Sebastian Bauer, Nadine Setzer, Florian Gaksch, Sandra Sühnel, Jason G. Skinner, Rickmer Braren, Irina Heid, Geoffrey J. Topping, Franz Schilling

**Affiliations:** ^1^ Department of Nuclear Medicine, TUM School of Medicine and Health, TUM University Hospital, Klinikum Rechts der Isar Technical University of Munich Munich Germany; ^2^ Department of Physics, TUM School of Natural Sciences Technical University of Munich Garching Germany; ^3^ Institute of Diagnostic and Interventional Radiology, TUM School of Medicine and Health, TUM University Hospital, Klinikum Rechts der Isar Technical University of Munich Munich Germany; ^4^ German Cancer Consortium (DKTK) Partner Site Munich and German Cancer Research Center (DKFZ) Heidelberg Germany; ^5^ Munich Institute of Biomedical Engineering Technical University of Munich Garching Germany

**Keywords:** ^13^C hyperpolarization, MR‐spectroscopy, pH, pyruvate, semi‐LASER

## Abstract

**Purpose:**

To develop and evaluate sequences for multi‐voxel magnetic resonance spectroscopy using hyperpolarized molecules.

**Methods:**

A standard single voxel PRESS sequence was extended to acquire multiple voxels consecutively. Its SNR was compared against a 2D FID‐CSI with both ^1^H and hyperpolarized ^13^C nuclei in phantoms and in a healthy mouse at 7T. This sequence was also used to determine tumor pH and metabolic activity in an endogenous murine pancreatic ductal adenocarcinoma model. Furthermore, a semi‐LASER sequence, using adiabatic full passage RF pulses for refocusing, was implemented. Multi‐voxel PRESS and semi‐LASER were then compared in healthy mice for measuring metabolic activity and pH using hyperpolarized [1‐^13^C]pyruvate and [1,5‐^13^C_2_]Z‐OMPD, respectively.

**Results:**

Multi‐voxel PRESS and semi‐LASER detected ^13^C metabolites in mouse kidneys and endogenous pancreatic ductal adenocarcinoma (PDAC) tumors with SNR comparable to that of standard 2D FID‐CSI. They enable fast MRS with a high spectral resolution that is highly customizable to recover spectra from regions not coverable by a single CSI slice.

**Conclusion:**

For the first time, we show hyperpolarized MRS using multi‐voxel PRESS and semi‐LASER sequences for hyperpolarized ^13^C‐labeled molecules. By implementing a semi‐LASER sequence using adiabatic full passage refocusing pulses, RF saturation was reduced. Semi‐LASER allows flexible overlapping of voxel refocusing planes, while for PRESS, signal from these regions is attenuated.

## 1 INTRODUCTION

Hyperpolarization (HP) allows the detection of low‐concentration ^13^C‐labeled molecules in vivo by increasing the SNR compared to the thermally polarized state by more than 10 000‐fold.^1^ Over the years, numerous applications using dissolution dynamic nuclear polarization (dDNP), in both animals and humans, have demonstrated the method's capability of non‐invasively determining tissue properties.^2^, ^3^, ^4^, ^5^

Most prominently, [1‐^13^C]pyruvate is employed to characterize metabolism in normal and diseased tissues.^6^, ^7^, ^8^, ^9^, ^10^, ^11^, ^12^ However, other applications using [^13^C]urea, [1,5‐^13^C_2_]Z‐OMPD, or [1,4‐^13^C_2_]fumarate have been investigated, to measure perfusion,^13^, ^14^ pH,^13^, ^15^, ^16^ or necrosis, respectively.^17^

One of the largest challenges in hyperpolarized MRI is the quick decay of hyperpolarized state in blood, which makes optimized pulse sequences an important topic of study in the field.^18^ About half of human applications to date have used metabolite‐selective‐excitation based sequences, while the remainder used MRS techniques.^8^ For high‐resolution localized MRS, most commonly single‐slice CSI sequences are used,^18^ particularly when the resonance frequencies are being measured, rather than being assumed to be known and constant during sequence design. In a pre‐clinical context, for example to measure pH, 2D FID‐CSI is often used.^13^, ^16^ However, specific cases require a more customizable type of sequence, especially when multiple target lesions cannot be covered by a single CSI slice, which is often the case in multifocal diseases such as metastatic pancreatic ductal adenocarcinoma (PDAC).^11^, ^19^

Point resolved spectroscopy (PRESS) and semi‐localization through adiabatic selective refocusing (semi‐LASER, sLASER) sequences are commonly used in high‐resolution MRS of the brain, due to their excellent spectral resolution and accurate localization.^20^, ^21^ Although these properties would also be highly desirable for hyperpolarized ^13^C MRS, to date, there are few publications known to the authors in which single‐voxel PRESS,^22^ LASER,^23^ or single‐voxel CPMG^24^ has been used for hyperpolarized ^13^C MRS, while the possibilities of semi‐LASER in this area have not been studied at all. In the present work, we developed and evaluated two fast and efficient sequences for hyperpolarized MRS: multi‐voxel PRESS (MV‐PRESS) and multi‐voxel semi‐LASER (MV‐sLASER). The sequences were evaluated at 7T in vitro and preclinically in vivo and were compared against 2D FID‐CSI. Finally, they were applied to measure tumor and kidney pH as well as metabolic activity in healthy and PDAC‐bearing mice.

## 2 METHODS

### 2.1 MR hardware

MR scans were performed on a 7 T preclinical MRI scanner (Discovery MR901 magnet and gradient system, Agilent, Santa Clara, USA; AVANCE III HD electronics, Bruker, Billerica, USA). For phantom measurements, a 31 mm ^1^H/^13^C dual‐tuned volume resonator (RAPID Biomedical, Rimpar, Germany) was used. Animal experiments were predominantly carried out using the same coil, however in certain cases, a 72 mm ^1^H/^13^C volume resonator (RAPID Biomedical) in combination with a ^13^C two‐channel surface receiver coil (RAPID Biomedical) was used, due to coil availability (Table S1).

### 2.2 Animal subjects

Eight Ptf1a^Cre/wt^;LSL‐KRAS^LSL‐G12D/wt^;Trp53^fl/fl^ mice^19^, ^25^ with endogenously grown PDAC tumors as well as three healthy C57BL/6 mice were anesthetized using 2% isoflurane/100% oxygen (2 L/min flow rate) and injected with 8.6 ± 1.2 mL/kg [1,5‐^13^C_2_]Z‐4‐methyl‐2‐oxopent‐3‐enedioic acid (Z‐OMPD) and 7.3 ± 2.6 mL/kg [1‐^13^C]pyruvic acid hyperpolarized solutions via a tail vein catheter. Rectal temperature and breathing were monitored during the time in the MRI. When animals received two injections, those were spaced apart by 1 h.

All experiments were conducted under the following approved animal licenses ROB‐55.2‐2532.Vet_02–18‐91 and ROB‐55.2‐2532.Vet_02–23‐70.

### 2.3 Hyperpolarization

For pyruvate injections, 22–24 mg 14 M [1‐^13^C]pyruvate (Merck, Darmstadt, Germany) was polarized together with 15 mM OX063 trityl radical (GE Healthcare, Waukesha, WI, USA) and 1 mM gadoteric acid (Dotarem, Guerbet, France) in a commercial d‐DNP system (Hypersense, Oxford Instruments, Oxford, United Kingdom) at 3.35 T/1.2 K/94.139 GHz for more than 45 min. The compound was then rapidly dissolved in heated buffer solution (80 mM TRIS, 80 mM NaOH, 0.1 g/L Na‐EDTA) to neutralize pH and reach an average pyruvate concentration of 80 mM.

For Z‐OMPD injections, 36–39.5 mg 7 M Z‐OMPD (in DMSO, with 25 mM OX063) were polarized in the same d‐DNP system using identical parameters, except a longer build up time of at least 90 min. Dissolution was performed in deuterated buffer saline to prolong T_1_ ^13^ (80 mM TRIS, 90 mM NaOH, 0.1 g/L Na‐EDTA, Z‐OMPD concentration 40–50 mM).

### 2.4 MR sequences

**FIGURE 1:**
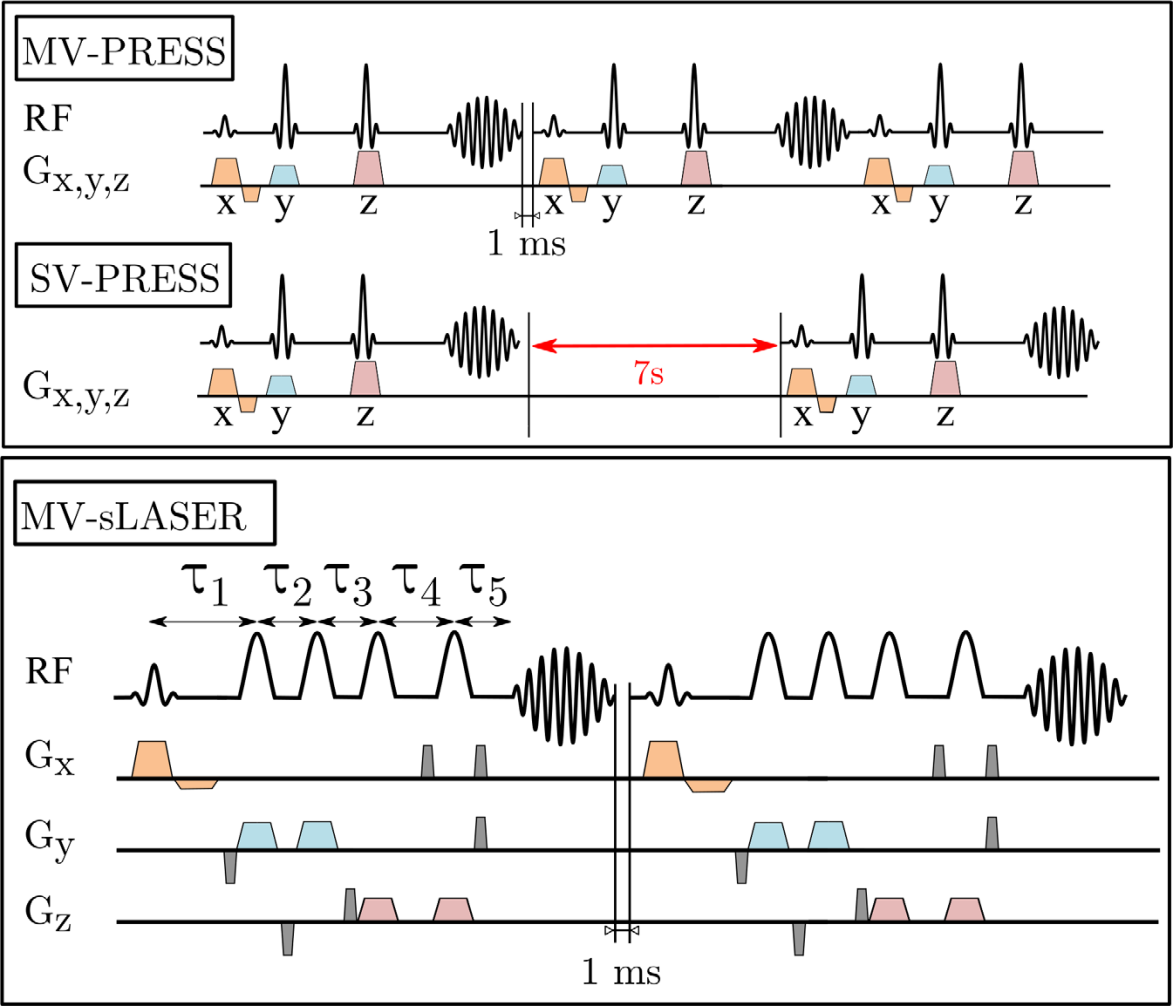
Multi‐voxel PRESS and multi‐voxel semi‐LASER sequence diagrams. For a train of stock single‐voxel PRESS sequences, a hardware delay of 7 s between scans makes them impractical for hyperpolarized MRS. The semi‐LASER sequence was adapted using adiabatic full passage refocusing pulses in HsN shape. Inter‐pulse TEs for semi‐LASER were adjusted to fulfill the condition 𝛕_1_ + 𝛕_3_ + 𝛕_5_ = 𝛕_2_ + 𝛕_4_. Slice selective gradients are shown in colors, while crushers are in gray. Gradient/RF heights and durations are not to scale. PRESS crushers around each RF pulse not shown for simplicity (see Figure S1 for a more detailed pulse sequence diagram).

One of the goals of this work was to set up voxel spectroscopy sequences with multiple voxels prior to an injection with a hyperpolarized ^13^C‐labelled substrate. To achieve this, one could set up stock PRESS voxels in the scanner interface (Paravision 6/7). However, on the MR system used in this work (Bruker AVANCE III), a non‐removable inter‐scan hardware delay of 7 s between successive sequences, related to the basic system electronics, renders this approach unfeasible for hyperpolarized acquisitions due to the non‐recoverable decay of magnetization. To circumvent this issue, a stock PRESS sequence was adapted to excite and read out multiple voxels consecutively (Figure 1). This was achieved by adjusting the sizes of the respective gradient and frequency matrices corresponding to slice‐selection. The resulting multi‐voxel PRESS sequence is able to excite another voxel immediately after the acquisition of the preceding echo has finished (see Figure S1 for a full sequence diagram). Furthermore, clarity of planning as well as postprocessing is improved if voxels are acquired within one sequence. RF pulses used in MV‐PRESS were standard calculated Shinnar‐Le Roux (SLR) pulses (sharpness = 3, bandwidth (90°‐/180°‐pulse) = 5.4/3.4 kHz, giving a chemical shift displacement error artifact of 11% for excitation and 26% for refocusing pulses relative to the slice thickness, for pyruvate and lactate, which are 900 Hz apart at 7T). TEs for PRESS and semi‐LASER were kept identical for comparisons (TE = 20.8 ms), while for comparison to CSI, PRESS TE was kept minimal (TE = 15.3 ms) to reduce T_2_(*) effects. Identical SLR excitation pulses were used for PRESS and semi‐LASER.

**FIGURE 2:**
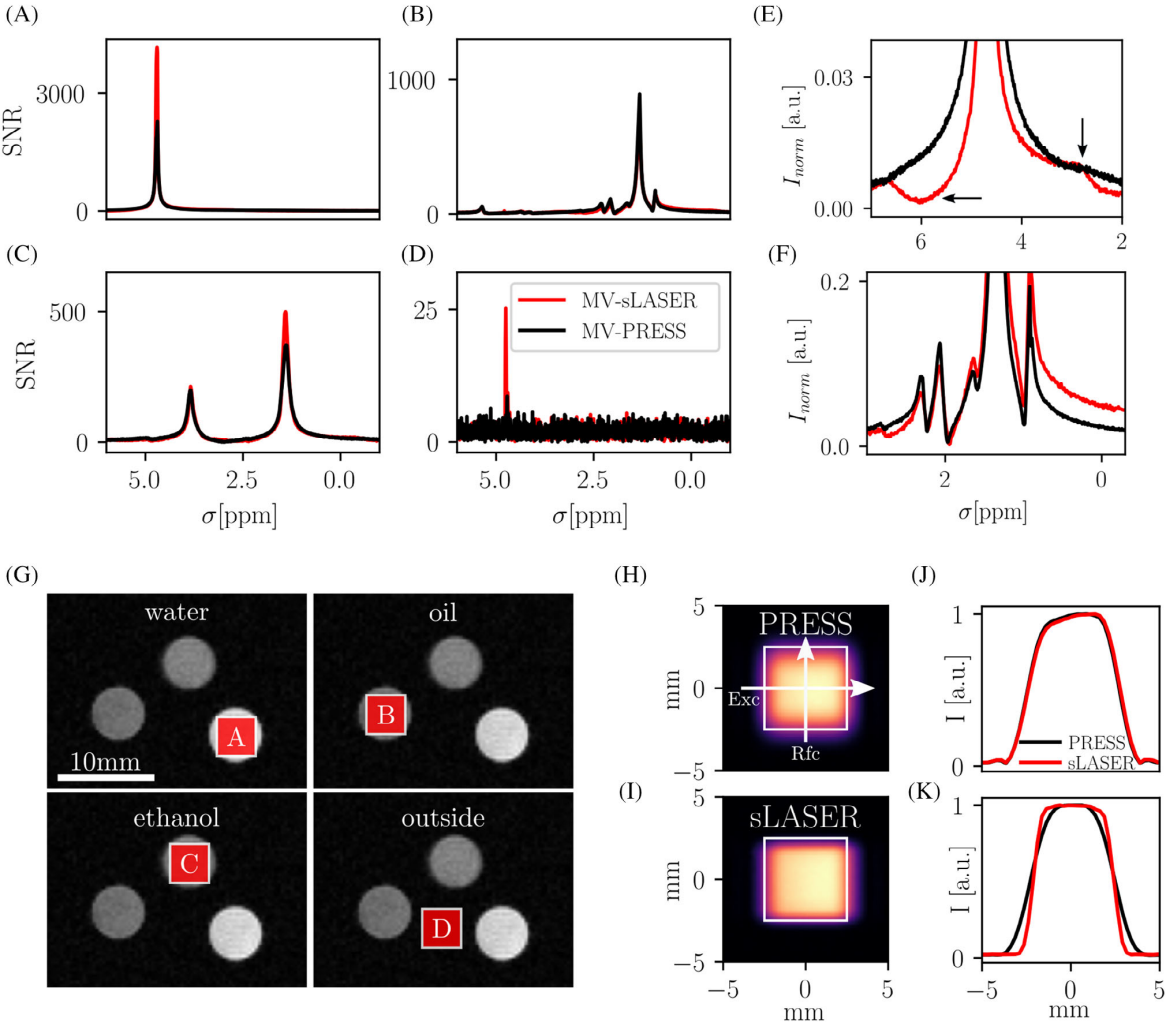
Multi‐voxel PRESS and semi‐LASER validation in ^1^H phantom containing water, oil, and ethanol tubes and PRESS and semi‐LASER slice profile measurements. Spectra acquired using the two multi‐voxel sequences are shown in (A)–(D) for the voxel locations displayed in (G) (spectra shown normalized to background noise). Exemplary zoomed‐in ROIs of maximum intensity normalized spectra in (A) and (B) are shown in (E) and (F) to highlight differences in signal between PRESS and semi‐LASER and artifacts from spurious echoes, which are indicated with arrows in (E). Slice profile measurements of excitation and refocusing directions for PRESS‐ and semi‐LASER‐based excitation pulse trains before a 2D FLASH readout are shown in (H) and (I). Line‐profiles along horizontal or vertical axes in (J), (K) highlight the sharper refocusing profile for semi‐LASER (K), while excitation profiles are identical (J). Prescribed voxels are highlighted as white boxes in (H), (I).

Since Paravision 6 and 7 do not feature stock semi‐LASER implementations, a custom semi‐LASER sequence was implemented based on the work by Scheenen,^20^ Landheer^21^ and Javed^26^ (Figure 1). Inter‐pulse timings were adjusted to fit the condition of 𝛕_1_ + 𝛕_3_ + 𝛕_5_ = 𝛕_2_ + 𝛕_4_ (Figure 1) for a total minimal TE of 20.8 ms. Crusher gradients around adiabatic full passage (AFP) RF pulses were adapted from Shams et al^27^ to follow a simple crushing scheme without phase cycling (relative gradient area values *G*_*x*_: 0, 0, 0, 1, 1; *G*_*y*_: −1, 0, 0, 0, 1; G_z_: 0, −1, 1, 0, 0; Figure 1). Crusher gradient peak amplitude was optimized experimentally (236 mT/m or 40% of maximal gradient strength), for a constant crusher duration of all crushers (0.5 ms), to minimize residual signal and spurious echoes while keeping spectral artifacts at high crusher strengths at a minimum (Figure 2E,F, see crusher optimization in Figure S2A–H). AFPs were generated using Pulse Wizard by Robin de Graaf^28^, ^29^ (HsN shape, RF constant c1 = 5.3, RF constant c3 = 1, duration = 3.4 ms, bandwidth = 3.2 kHz) and then imported into Paravision 7 (see Figure S2K–L). Chemical shift displacement artifacts due to excitation are identical to PRESS and for refocusing are slightly larger due to the lower bandwidth (28% for pyruvate and lactate). Necessary peak B_1_ strength for AFP refocusing pulses was determined to be 0.04 mT for ^1^H and 0.21 mT for ^13^C, through a series of inversion recovery sequences with varying RF power for the AFP (Figure S2I–J).

To measure the slice profile of PRESS and semi‐LASER in the excitation and refocusing directions, a FLASH sequence was modified to use the slice selective gradients and RF pulses from a PRESS or semi‐LASER sequence as excitation, without spectral readout. Then, 2D‐FLASH readout was carried out to provide images of the excited voxels (Figure 2H,I) as well as line plots in excitation and refocusing direction (Figure 2J,K).

Voxels for both sequences were placed using an in‐house developed tool to monitor and avoid excitation and refocusing plane overlap (Figure S3).

### 2.5 Thermal phantoms

Sequences were validated in ^1^H phantoms consisting of three 5 mm glass NMR tubes filled with either water, ethanol, or canola oil. AFP pulse power and PRESS and semi‐LASER slice profiles were determined using a 50 mL Falcon tube phantom filled with water, 2 mM Gd‐DOTA contrast agent, and 2 M ^13^C‐labeled GMP‐grade urea.

### 2.6 Hyperpolarized phantoms

For hyperpolarized in vitro experiments, a 3D printed hollow sphere phantom (1 mL) was first filled with water for correct positioning, reference images, voxel placement, and adjustments and then emptied before injection with the same volume of hyperpolarized liquid. An Eppendorf tube filled with [1‐^13^C]‐labeled 2.2 M lactate and 2 mM Gd‐DOTA was taped to the side of the sphere phantom for ^13^C power adjustment. The phantom was placed onto an animal bed with spacers taped to the bed to ensure proper positioning of the phantom after removal for filling with hyperpolarized substrate. Anatomical references images were taken after each dissolution and re‐insertion of the sphere into the bore to ensure accurate positioning of prescribed voxels.

### 2.7 MV‐PRESS vs. CSI SNR comparison

The SNR of the MV‐PRESS sequence (in vitro: TE/TR = 15/480 ms, acquisition points = 1024, receiver bandwidth = 30 ppm, voxel size = (2 mm),^3^ spectral resolution = 1.1 Hz/pt.; in vivo: TE/TR = 17/1000 ms, acquisition points = 1024, receiver bandwidth = 40 ppm, voxel size = (2 mm),^3^ spectral resolution = 1.5 Hz/pt.) was compared to a standard 2D FID‐CSI sequence in vitro (matrix size = 12 × 8, acquisition points = 512, flip angle = 10°, voxel size = 2 × 2 mm^2^, spectral resolution = 2.2 Hz/pt., slice thickness = 2 mm, scan time = 22.1 s, TR/TE = 230/1 ms, receiver bandwidth = 30 ppm, centric‐centric k‐space encoding starting from center of k‐space, see Figure S4) and in vivo (matrix size = 13 × 11, acquisition points = 256, flip angle = 12°, voxel size = 2 × 2 mm^2^, spectral resolution = 5.9 Hz/pt., slice thickness = 2 mm, scan time = 12.9 s, TR/TE = 90/2.7 ms, receiver bandwidth = 40 ppm). RF pulse bandwidths for excitation were CSI 5 kHz and PRESS: 5.4 kHz. To monitor variations in resulting signal due to injection time and polarization level, each acquisition was preceded by a small flip‐angle unlocalized spectroscopic acquisition, starting 4 s after the injection ended (flip angle = 2°, acquisition points = 2048, receiver bandwidth = 40 ppm). The MRS sequences were acquired 8 s after the unlocalized acquisition due to the 7 s hardware delay between successive sequence acquisitions and the acquisition time of the unlocalized spectrum.

SNR was computed for each magnitude spectrum peak as the ratio of (baseline corrected) signal level to background region standard deviation. Signal level for each spectral peak was defined as its fitted height. Background spectral regions were those without peaks present, usually at the edges of the spectrum. This was verified by calculating apparent background SNR, using the mean of the magnitude spectrum as the signal level, which was consistently approximately 2, which is also roughly consistent with Rayleigh distributed noise.

### 2.8 Data analysis

Data were analyzed using custom‐written Python 3.10 scripts and standard packages (numpy, scipy, matplotlib, pandas). The spectroscopic raw data were fit in time‐domain by iteratively minimizing the sum‐of‐squares of the difference of the measured data and the modeled FID data. Fitting was needed to precisely determine peak frequencies for pH computation and peak amplitudes for lactate to pyruvate ratios and area under the curve plots. Example analysis Jupyter Notebooks and the custom python package are found on Github (https://github.com/Schilling‐Lab/2024‐mv‐press‐slaser‐publication.git).

For figures, ^13^C spectra were linebroadened by 5 Hz for improved visualization of metabolite peaks.

## 3 RESULTS

### 3.1 Sequence validation

The MV‐PRESS and semi‐LASER sequences were compared against a stock PRESS sequence in a three‐tube phantom containing water, oil, and ethanol, to validate the correct positioning of voxels. MV‐PRESS and stock PRESS provide identical spectra (see Figure S5); therefore, only the comparison of the multi‐voxel versions of semi‐LASER and PRESS is shown in Figure 2A–D for the positions shown in Figure 2G (zoomed in regions of spectra in Figure 2A,B shown in Figure 2E,F). Spurious echoes are minimized through experimentally determined crusher strengths (see Figure S2). Slice excitation and refocusing profiles of PRESS and semi‐LASER were investigated for different voxels sizes from (2–10 mm)^3^ with an exemplary (5 mm)^3^ voxel shown in Figure 2H,I, and line plots corresponding to excitation and refocusing directions (Figure 2J,K). Excitation profiles are identical for both sequences (Figure 2J), while the refocusing profile of semi‐LASER is sharper than for PRESS (Figure 2K), as expected from literature.^28^

### 3.2 SNR comparison of MV‐PRESS with 2D FID‐CSI

**FIGURE 3:**
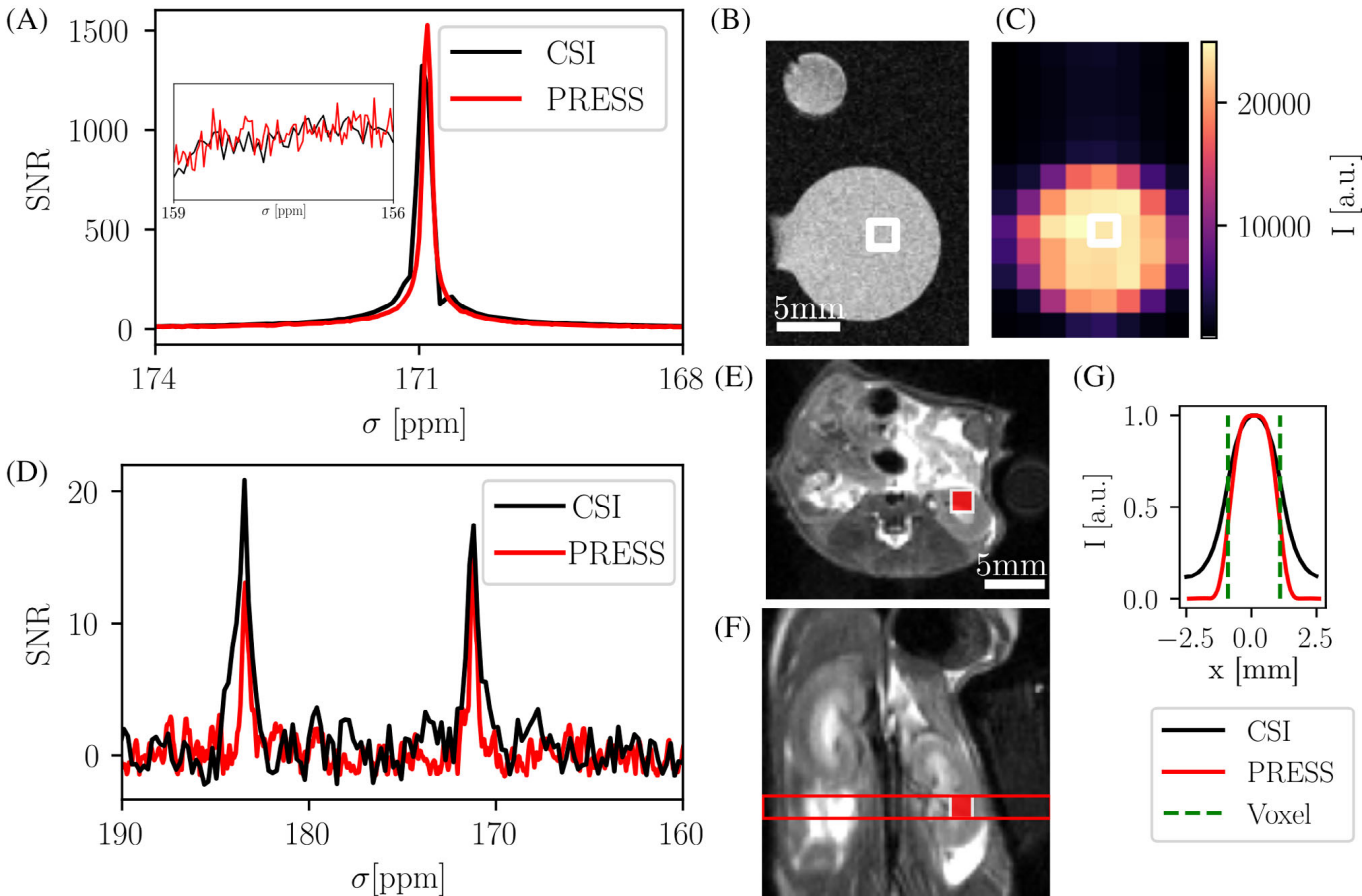
SNR comparison of multi‐voxel PRESS and 2D FID‐CSI using hyperpolarized [1‐^13^C]pyruvate in vivo and in vitro. (A) Spectra of identically prescribed voxels from CSI and PRESS in spherical phantom showing similar SNR for the sequences (CSI: 1321 vs. PRESS: 1525), with an inset of the background region near 157 ppm used for SNR calculation shown. (B) Proton reference image of 3D printed spherical phantom with adjacent thermally polarized [1‐^13^C]lactate in an Eppendorf tube. (C) The 2D FID‐CSI intensity map with voxel position shown in white. (D) Spectra from a mouse kidney voxel showing both pyruvate and lactate, acquired with PRESS or 2D FID‐CSI after separate injections of HP PA. (E, F) Axial/coronal T_2_w reference images showing slice and voxel placement. (G) Signal bleeding in the image plane due to the point spread function of 2D FID‐CSI with an in plane resolution of 2 mm compared to the simulated refocusing pulse profile of a 2 mm PRESS voxel (see also Figure S4).

The MV‐PRESS sequence was compared against a 2D FID‐CSI in a hyperpolarized [1‐^13^C]pyruvate phantom as shown in Figure 3A–C. Hyperpolarized solution was injected into a 3D printed sphere twice within one MR session, once for a CSI acquisition and again for a PRESS acquisition. Spectra from the two voxels are shown in Figure 3A. Peak SNR is similar between the sequences (SNR_CSI_ = 1321, SNR_PRESS_ = 1525). Total signal from preceding low‐flip‐angle unlocalized spectra was equivalent (˜1%). Figure 3C shows an SNR map of the CSI slice with the comparison voxel highlighted in white. The experiment was repeated with similar results (SNR_CSI_ = 1885, SNR_PRESS_ = 2224, where the polarization level for the CSI acquisition was lower by a factor of 0.85). Computed pyruvate‐hydrate to pyruvate ratios are similar (CSI/PRESS = 0.07/0.08).

Furthermore, SNR was compared in vivo in the kidney of a PDAC mouse (Figure 3D–F). An identical kidney voxel was targeted first by MV‐PRESS and later by 2D FID‐CSI, resulting in the spectra shown in Figure 3D. T_2_w anatomical reference images showing CSI slice placement and PRESS voxel location are displayed in Figure 3E,F. SNR for pyruvate and lactate between the two sequences is similar (pyruvate: SNR_CSI_ = 17, SNR_PRESS_ = 16, lactate: SNR_CSI_ = 21, SNR_PRESS_ = 13). Lactate to pyruvate ratios are comparable (CSI/PRESS = 1.0/0.9). Total signal from preceding low‐flip‐angle unlocalized spectra was a factor 1.1 higher for the CSI acquisition. Expected spatial bleeding of signal due to the point spread function (Figure 3G) of the CSI is additionally highlighted using simulations in Figure S4.

### 3.3 MV‐PRESS for in vivo pH detection in PDAC

**FIGURE 4:**
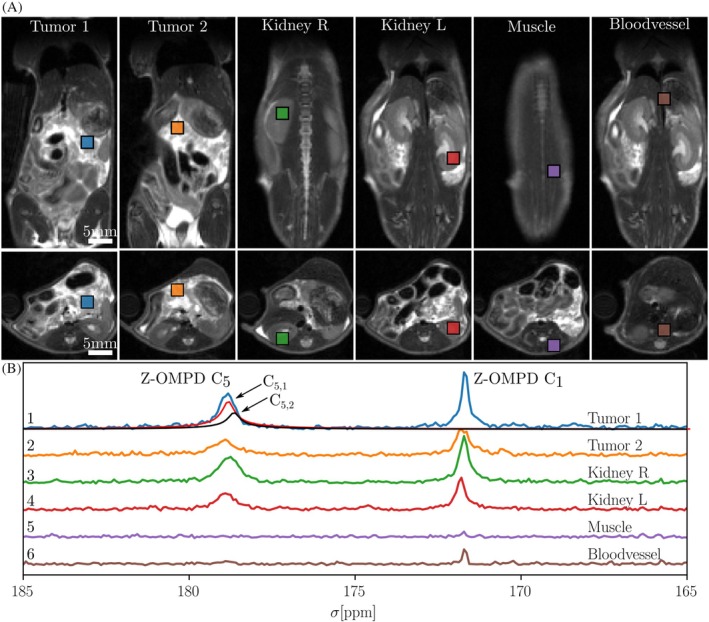
In vivo pH‐measurement of PDAC and kidneys in mice using MV‐PRESS. Anatomical references of animal 1 are shown in (A) for 6 voxels (sized 2.5 × 2.5 × 2.5 mm^3^). Spectra from MV‐PRESS in (B) show Z‐OMPD resonances on C_1_ and C_5_ with a SNR above the noise background (SNR = 3–4). Muscle and artery voxels show low SNR due to overlap of previous excitation or refocusing planes as well as sub‐optimal shims or low perfusion. pH and SNR values are given in Table S2. Multi‐compartment fitted spectra for the splitted C_5_ resonances (C_5,1_ and C_5,2_) due to extracellular extravascular tumor acidification are shown as red and black lines, as published previously.^13^

In vivo pH was determined in two animals bearing endogenous PDAC using MV‐PRESS and HP Z‐OMPD (animal 1 shown in Figure 4, animal 2 in Figure S6). Voxels were placed in tumor nodules, kidneys as well as muscle tissue and a blood vessel, showing both Z‐OMPD C_1_ and C_5_ peaks (Figure 4B) with a high SNR (SNR (C_1_,C_5_) = 38/16 in Tumor 1, see Table S2). Animal 1 showed overall higher SNR as well as lower pH values, hinting at acidification due to the PDAC or due to respiratory acidosis during anesthesia.^30^, ^31^ Scan parameters for both animals are shown in Table S1. Six PDAC animals imaged during the first phase of this study using HP PA and MV‐PRESS are shown in Figure S7 with anatomicals in Figure S8.

### 3.4 Comparison of MV‐PRESS and MV‐semi‐LASER in healthy mice

**FIGURE 5:**
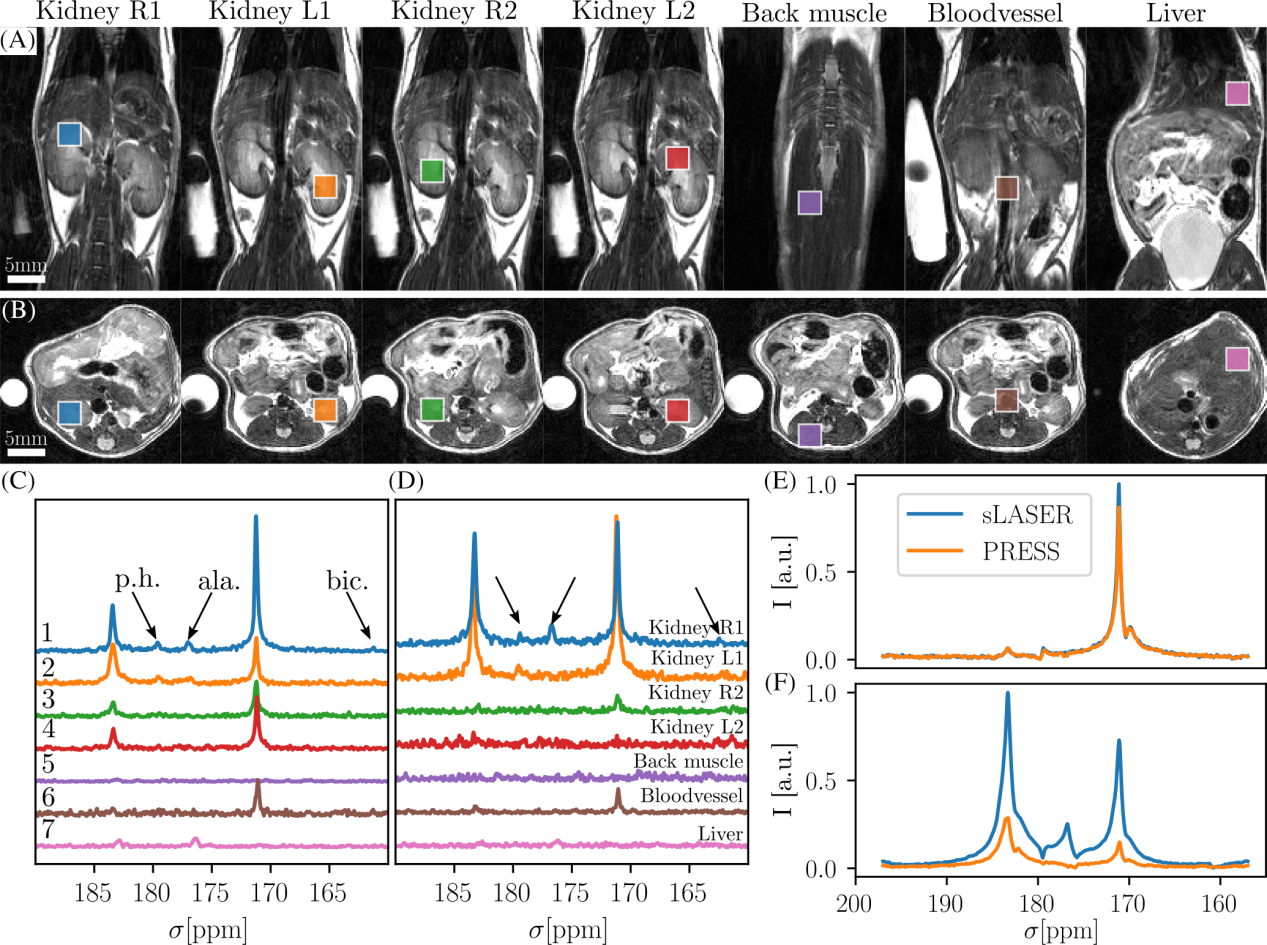
Comparing multi‐voxel PRESS and semi‐LASER in a healthy mouse using hyperpolarized [1‐^13^C]pyruvate. Seven voxels were placed in different organs/regions (A, B) T_2_w anatomical references, coronal and axial, with voxel locations overlayed. (C, D) Semi‐LASER/PRESS spectra with low‐signal metabolites pyruvate‐hydrate (p.h.), alanine (ala.), and bicarbonate (bic.) highlighted. Spectra are shown line‐broadened (5 Hz) and normalized to background noise. (E) 1° full‐volume excitation 6/7 s after start of injection and 8 s before start of semi‐LASER/PRESS acquisition. (F) The 90° non‐selective excitations after MRS, showing that MV‐semi‐LASER left three times more hyperpolarized magnetization than MV‐PRESS.

To compare semi‐LASER to PRESS in a static acquisition, a healthy mouse was injected twice with HP PA (Figure 5A,B). Differences between the injections in polarization level and injection volume and resulting overall signal level are below 5% relatively between acquisitions, computed from an initial low flip angle non‐selective excitation (Figure 5E) that was started 6–7 s after start of injection. This timing was chosen to maximize lactate signal in the MRS spectra, which were acquired 9 s later. Spectra from semi‐LASER show a similar SNR to that of PRESS (Kidney L1: SNR_Pyr,sLASER_ = 77 vs. SNR_Pyr,PRESS_ = 33, SNR_Lac,sLASER_ = 22 vs. SNR_Lac,PRESS_ = 25) in the first voxels (Figure 5C,D). In subsequent voxels, PRESS signal is close to the background noise (Kidney L2: SNR_Pyr,sLASER_ = 12 vs. SNR_Pyr,PRESS_ = 3) due to overlap of refocusing and excitation slices with previously acquired voxels, which causes saturation or residual inversion of the hyperpolarized magnetization by RF excitation or refocusing (Kidney L2 overlaps with Kidney L1). The sequence was set up with 10 voxels (3 mm)^3^ to evaluate the possibility of additional signal; however, only the first 7 voxels exhibited signals above noise level and are therefore displayed here. In total, semi‐LASER is able to detect metabolites in six of seven voxels with SNR above noise level, and PRESS in five of seven with an apparent SNR reduction (see Table S3 for SNR values for all metabolites). A non‐selective excitation after MRS shows that semi‐LASER leaves three times more ^13^C polarization intact than does MV‐PRESS (Figure 5F) in this configuration. Lactate to pyruvate ratios differ between semi‐LASER and PRESS spectra (voxel 1: 0.4 vs. 1.1, voxel 2: 1.6 vs. 0.7), see Table S4. Full width at half maximum (FWHM) values for fitted spectra are shown in Table S5. The experiment was repeated twice (Figures S10, S11, Tables S6–S9), where PRESS SNR is comparable or even higher than semi‐LASER in the first voxel, but then decreases for following voxels with overlap in previous excitation/refocusing planes.

**FIGURE 6:**
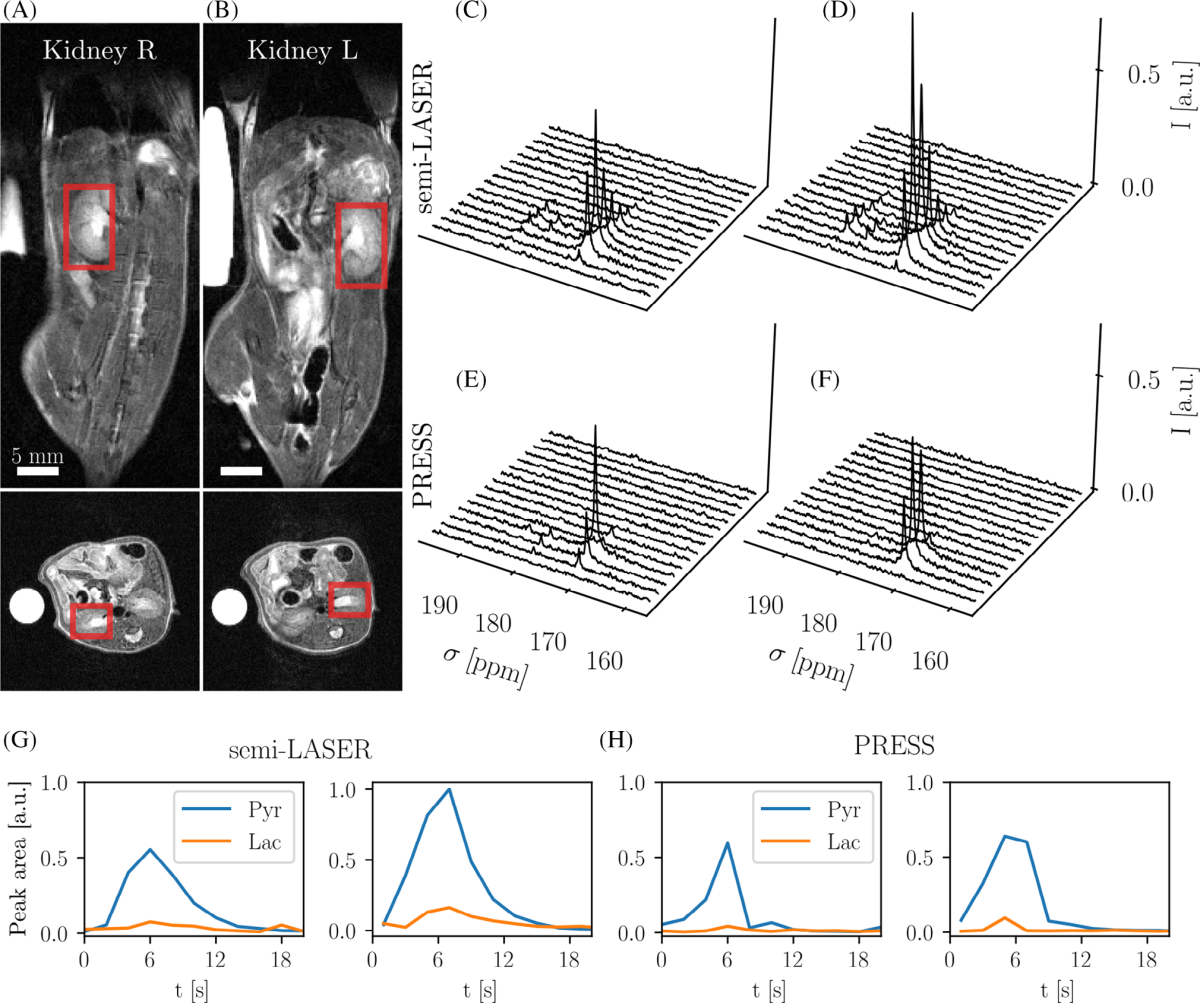
Dynamic multi‐voxel PRESS and semi‐LASER in healthy mouse kidneys using hyperpolarized [1‐^13^C]pyruvate. (A, B) Anatomical T_2_w reference images of both kidneys and voxel placement. (C, D) Semi‐LASER spectra from left and right kidney, respectively. (E, F) PRESS spectra from left and right kidney. The semi‐LASER spectra have higher pyruvate peaks and also resolve more metabolite peaks at higher ppm offsets from the main pyruvate peak, including lactate, than do the PRESS spectra. (G, H) Peak area time curves from spectral fits of pyruvate and lactate. Effective T_1_ computed for pyruvate peak intensities is reduced three‐fold for PRESS spectra (1 vs. 3–4 s for semi‐LASER spectra). Spectra are shown line‐broadened and normalized to the maximum intensity in semi‐LASER spectra. The semi‐LASER dissolution had a 1.13‐fold higher polarization level.

Next, the dynamic capabilities of semi‐LASER and PRESS (for both: excitation FA = 35°, TR = 2 s, voxel size = 5.5 × 4.5 × 9.7 mm^3^) were assessed in the two kidneys of a healthy mouse (Figure 6). Two voxels covered one kidney each and overlapped in the refocusing directions, but not in the excitation direction (Figure 6A,B). Spectra from semi‐LASER show lactate and pyruvate at close to 3× higher SNR (maxima: SNR_Pyr,sLASER_ = 98, SNR_Pyr,PRESS_ = 35, SNR_Lac,sLASER_ = 5, SNR_Lac,PRESS_ = 2, Figure 6C,D) than that of PRESS (Figure 6E,F). The polarization level for the semi‐LASER dissolution was 1.13‐fold higher than that for PRESS. Peak area time curves extracted from spectroscopic fits for pyruvate and lactate peak amplitudes show pyruvate to lactate conversion (Figure 6G,H). Effective T_1_ of pyruvate is reduced for PRESS spectra compared to semi‐LASER (0.7–1.0 s vs. 2.7–3.8 s). A more extensive quantitative comparison between time curves for PRESS and semi‐LASER remains difficult due to the low signal in PRESS spectra. A repeated experiment is shown in Figure S12, similarly showing an improved SNR of metabolites for the semi‐LASER acquisition.

**FIGURE 7:**
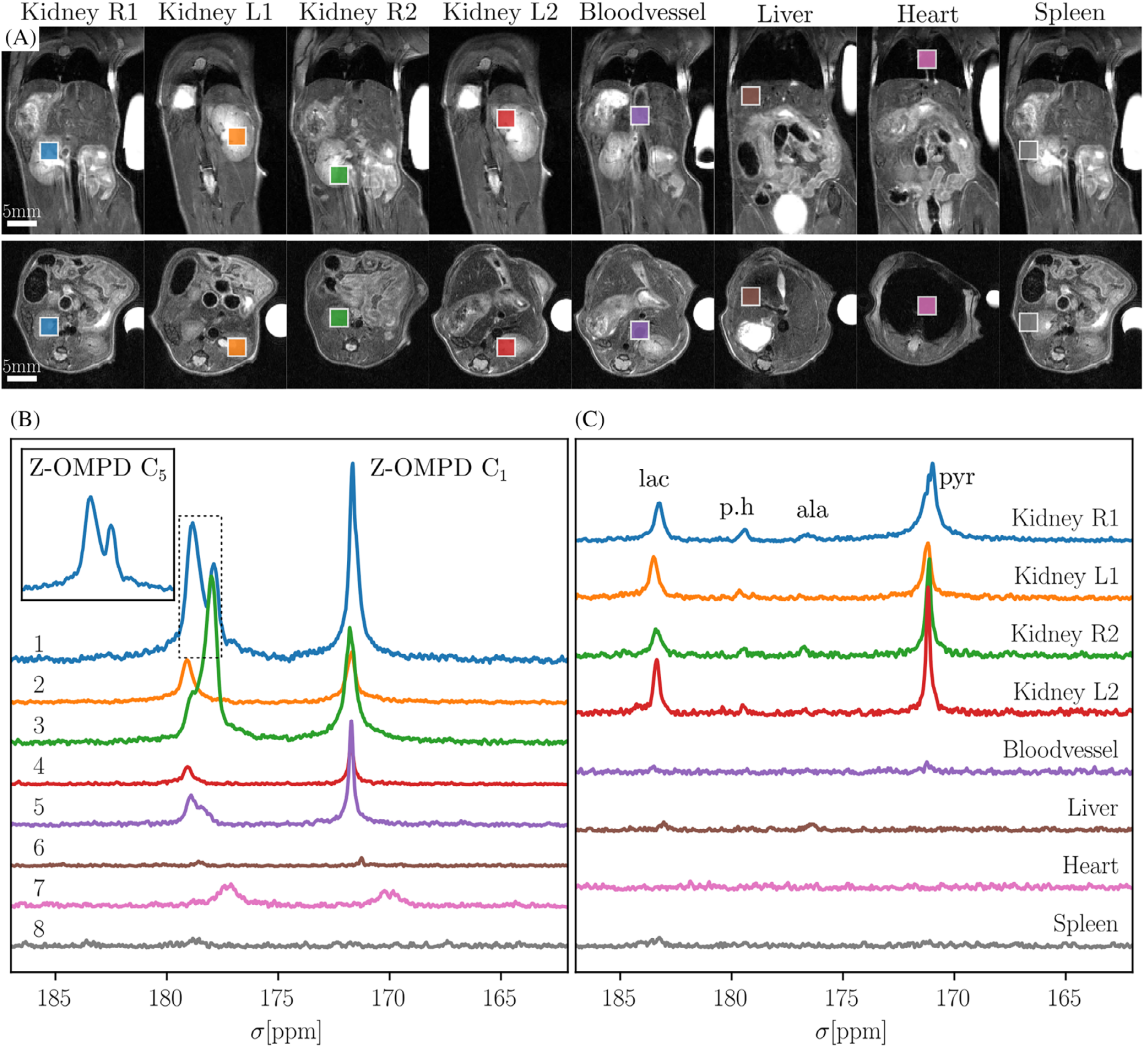
Detecting pH and metabolism in healthy mouse kidneys using [1,5‐^13^C_2_]Z‐OMPD and [1‐^13^C]pyruvate. (A) Coronal and axial T_2_w anatomical scans showing voxel placement. (B) Spectra acquired from MV‐semi‐LASER after injection with HP Z‐OMPD. Note peak splitting of the C_5_ peak at 179 ppm due to multiple pH compartments, see inset in B. (C) Spectra obtained from MV‐semi‐LASER after injection with HP PA, 1 h later. PH and SNR values from Z‐OMPD spectra for a three‐compartment fit are shown in Table S10.

Finally, healthy kidney pH was determined using HP Z‐OMPD and the multi‐voxel semi‐LASER sequence in eight voxels (Figure 7A,B, acquisition started 10 s after end of injection). Results from (3 mm)^3^ voxels show Z‐OMPD‐C_1_ and C_5_ peaks at a high SNR with peak splitting due to pH compartmentation (Table S10), as reported previously.^13^ Multi‐compartment fits result in pH values corresponding to cortex (pH = 7.32 ± 0.02), medulla (pH = 7.06 ± 0.01) and pelvis (pH = 6.58 ± 0.01), in line with previous measurements.^13^, ^16^, ^32^ Additionally, a second injection with HP pyruvate shows pyruvate, lactate, pyruvate‐hydrate, and alanine signal in both kidneys, a central blood vessel and the liver (acquisition started 8 s after end of injection).

## 4 DISCUSSION

The multi‐voxel versions of PRESS and semi‐LASER implemented here are able to acquire localized high‐resolution spectra for hyperpolarized ^13^C MRS within a short period of time compared to the lifetime of the hyperpolarized state in vivo. Over the course of the study, many voxel sizes and total numbers were investigated for preclinical mouse imaging. The main tradeoff that needs to be made is between voxel number and size, since both potentially lead to overlap in excitation and refocusing slices which should be avoided due to irreversible RF saturation of the hyperpolarized signal. Larger voxels will increase the signal, but lead to either a low amount of selected voxels or unavoidable slice overlap. Signal loss by overlap can be reduced with the use of adiabatic refocusing pulses (effectively semi‐LASER), as shown here, but still skews the results since part of the voxel has already experienced some excitation or refocusing. Therefore, pH values from voxels with slice(s) overlapping previously acquired voxels are weighted toward the non‐excited/refocused tissue regions. Smaller voxels lead to low SNR, due to the inherently small volume being excited (for example, a (2 mm)^3^ voxel, which encompasses a PDAC tumor nodule, only collects signal from 8 μL of tissue).

For non‐dynamic applications, these sequences can be used to acquire local spectroscopic data to determine, for example, pH or metabolite ratios. Dynamically (excitation FA = 35°), the semi‐LASER variant is able to detect spectra from two ROIs with an increased SNR compared to PRESS, due to the use of adiabatic refocusing pulses, which are more robust to B_1_ imperfections and better preserve magnetization for multiple excitations.^33^ RF saturation is an issue in both sequences and, while semi‐LASER is able to alleviate this partially, compared to slice‐selective MRS or non‐localized spectra, effective T_1_ is still reduced drastically (PRESS: 1 s, semi‐LASER: 3–4 s vs. slice selective spectroscopy: 10 s^34^).

The SNR of static PRESS (excitation FA = 90°) in vitro and in vivo [1‐^13^C]pyruvate and lactate spectra were similar to those of a standard static 2D FID‐CSI (FA = 10° or 12°). An advantage of single voxel MRS compared to a 2D FID‐CSI sequence is a reduced signal bleeding artifact. As seen in phantom experiments (Figure 3) and simulations (Figure S4), the point‐spread‐function of the 2D FID‐CSI leads to bleeding of signal between the target voxel and neighboring regions due to signal weighting in k‐space due to T1 and RF saturation. This leads to distorted measurements, since, for example, the often‐large signal peak from a central blood vessel will bleed into neighboring regions, skewing the resulting spectra. Single voxel MRS with sharp excitation and refocusing profiles, as shown here, is not prone to this effect.

Additionally, in cases where multiple FID‐CSI slices are needed, loss of signal in subsequent slices is inevitable, due to the decay of hyperpolarized signal during the longer of the CSI sequence. For example, a three slice FID‐CSI, covering approximately the same seven voxels as highlighted in Figure 7, would take about 39 s to acquire, while the multi‐voxel sequence used here can be acquired in less than 7 s, with sequence parameters otherwise unchanged. Furthermore, the additional information from slice acquisitions may not be needed, for example if a tumor nodule needs to be characterized that is far away from the rest of the ROI. Here, the customizable multi‐voxel versions of PRESS or semi‐LASER can provide efficient spectral acquisitions covering multiple independent voxels that are spread across a 3D volume but are not within a single plane. A limitation, however, is the time consuming positioning of voxels, to ensure correct locations and minimal slice overlap, which must be performed separately for each subject and session.

Ideally, each voxel would be shimmed separately and adjusted dynamically in between acquisitions. However, in this work, B_0_ map‐based local shims of the entire mouse abdomen were the best available option due to hardware limitations. This leads to peak broadening and signal loss in badly shimmed regions (see Figure S9), such as muscle tissue in the legs or at the back, as well as the heart. Better dynamic and active shimming^35^, ^36^ could substantially increase the SNR as well as number of possible voxel locations.

Chemical shift displacement artifacts due to RF bandwidth and multiple resonances is similar between MV‐PRESS and MV‐semi‐LASER sequences in this work (excitation: 17%, refocusing: 26% (PRESS) or 28% (semi‐LASER), for pyruvate and lactate). For semi‐LASER this could be improved by using larger bandwidth refocusing pulses, which, in order to maintain the adiabatic condition, is limited by the available RF maximum peak amplitude of the setup.

For this work, all excitation and refocusing slices placed for multi‐voxel MRS sequences were scanner axis aligned as well as of the same thickness. Future work could enable voxels of variable sizes and orientations, as well as minimal overlap between excitation and refocusing planes by enabling angulated slices. A more extensive positioning software, possibly based on automatic organ and tumor segmentation could further improve the number, size and positioning of voxels and aid in decreasing the setup time.

Translation of this sequence to a clinical setting could be performed in a similar fashion, by expanding the respective gradient and frequency arrays. However, other manufacturers might not need this extension in case there is no hardware dead‐time in between sequences present. For the SVS sequence, previous work has shown that semi‐LASER is able to collect higher‐quality spectra from the human brain at 7 T than PRESS (B_1_ = 0.03 mT). Especially if surface coils, which generally lead to a higher SNR close to the coil, are used (Tx,Rx), the adiabatic refocusing pulses lead to an increased robustness against B_1_ inhomogeneities.^37^, ^38^ Finally, the smaller gyromagnetic ratio of ^13^C requires higher RF amplitudes compared to ^1^H potentially increasing TE due to longer RF pulses.

## 5 CONCLUSIONS

Localized MRS sequences, such as PRESS and semi‐LASER, are used widely in proton MRS, but have so far not been tested in detail for hyperpolarized ^13^C MRS. Here, we showed their applicability for hyperpolarized ^13^C MRS preclinically using [1‐^13^C]pyruvate and Z‐OMPD to determine tumor/kidney metabolic activity and pH, while acquiring signal from up to eight voxels within 5 s and at high spectral resolution. Healthy and PDAC mouse experiments showed the applicability of the sequences for pH detection, similar to a non‐invasive in vivo virtual 3D biopsy.

## CONFLICT OF INTEREST STATEMENT

Franz Schilling serves on the scientific advisory board of NVision Imaging Technologies GmbH.

## Supporting information


**Table S1:** PDAC MV‐PRESS mouse scan parameter **overview**. Coil 1 refers to 31 mm ^1^H/^13^C volume coil and coil 2 to 72 mm ^1^H/^13^C volume coil with ^13^C receiver array.
**Figure S1:** Sequence diagrams of single‐voxel PRESS, multi‐voxel PRESS and multi‐voxel semi‐LASER with crusher gradients for PRESS sequences.
**Figure S2:** Semi‐LASER crusher strength and adiabatic full passage RF‐pulse characteristics. In A‐G, magnitude spectra from a semi‐LASER voxel in an oil phantom are shown for varying crusher strengths in percent of the maximal gradient strength (590 mT/m). When compared to a PRESS spectrum (H), spurious echoes around 7–8 ppm at low crusher strengths are apparent (arrows in A–D). With increasing crusher strength, these echoes disappear but wave‐like artifacts around 3 and 7 ppm appear (arrows E‐G). Around 30%–40% of crusher strength both artifacts and spurious echoes are minimized. In I and J, the power for an AFP pulse was increased in an inversion recovery experiment to see where the pulse reaches inversion. For the proton channel (I), this happens at around 10 W (0.04 mT), while for carbon, 25 W (0.21 mT) is needed (J). K and L show the amplitude and phase of the 3.4 ms AFP HsN pulse used in this work.
**Figure S3:** Multi‐voxel MRS planning tool overview. Anatomical reference images can be loaded and voxel position as well as slice locations overlaid for accurate overlay monitoring.
**Figure S4:** 2D FID‐CSI point spread function and bleeding artifact simulation for a square shape. (A) K‐space encoding scheme for 2D FID‐CSI: each vertical column, as shown, of k‐space points is acquired in an up‐down alternating center‐out pattern, and then each horizontal row is acquired in a similar ordering left–right, giving substantially larger differences in remaining signal between k‐space points adjacent horizontally than vertically (B) Sample image of a uniform intensity square shape used for simulation of spatial bleeding and point spread function. (D) Convolution of point spread function and test image, with signal spatial bleeding visible in left–right direction, giving effective spatial resolution worse than the nominal voxel size and spacing, also highlighted in the subtraction of images (B) and (C) shown in (D).
**Figure S5:** Validation of multi‐voxel PRESS against stock single‐voxel PRESS sequence in a three‐tube phantom. Spectra at four locations (A: water, B: oil, C: ethanol, D: outside reference) were acquired using a single‐voxel PRESS sequence (black lines) as well a a four‐voxel MV‐PRESS (colored lines) sequence at the same locations (T_1_w proton reference image shown in E). Spectra are virtually identical, as expected.
**Figure S6:** In vivo pH measurement of PDAC animal 2 using Z‐OMPD and MV‐PRESS. In A, the six voxel ROIs are shown. Note that the third voxel (Kidney L) is within an unusually bright area in the kidney, possibly due to kidney blockage related to the PDAC. No signal is recorded in this ROI, in contrast to the other kidney (see spectra in B). C: pH values and SNR of OMPD peaks from multi‐compartment fit.
**Figure S7:** Static detection of [1‐^13^C]pyruvate and metabolites in PDAC mice using MV‐PRESS. Spectra acquired using MV‐PRESS for two setups used (A: 31 mm ^1^H/^13^C volume coil, B: 72 mm ^1^H/^13^C volume coil with ^13^C receiver array, see Table S1). Lactate to pyruvate ratios for tumor lesions shown in C. Anatomical references with voxel positions of respective animals are shown in Figure S8. Reference voxel data acquired in kidneys, muscle tissue and other organs not shown here.
**Figure S8:** Static detection of [1‐^13^C]pyruvate and metabolites in PDAC mice using MV‐PRESS. Anatomical references for tumor voxel spectra shown in Figure S6. (A) Animal 1,1. (B) Animal 1,2. (C) Animal 2. (D) Animal 3,2. (E) Animal 3,1. (F) Animal 4. (G) Animal 5. (H) Animal 6.
**Figure S9:** Proton reference spectra at voxel locations of the animal shown in Figure 7. Full width at half maximum (FWHM) values for the eight voxels range from 58 to 177 Hz at 7 T (83, 76, 118, 58, 69, 177, None, and 127) for (Kidney L1, Kidney R1, Kidney L2, Kidney R2, Artery, Liver, Heart and Spleen). Proton spectrum from the heart voxel is too low to compute a FWHM.
**Table S2:** In vivo pH values obtained using MV‐PRESS and Z‐OMPD for PDAC mouse shown in Figure 4. Uncertainties are obtained from fit accuracy.
**Table S3:** SNR values for comparison of multi‐voxel PRESS and semi‐LASER in a healthy mouse using hyperpolarized [1‐^13^C]pyruvate. Values for Figure 5.
**Table S4:** Lactate to pyruvate ratios for comparison of multi‐voxel PRESS and semi‐LASER in a healthy mouse using hyperpolarized [1‐^13^C]pyruvate. Values for spectra shown in Figures 5, S10, S11 for voxels with an SNR above noise background threshold in both sequences.
**Table S5:** FWHM values for comparison of multi‐voxel PRESS and semi‐LASER in a healthy mouse using hyperpolarized [1‐^13^C]pyruvate. Values for spectra shown in Figure 5 for voxels with an SNR above noise background in both sequences, as shown in Table S3.
**Figure S10:** Comparing multi‐voxel PRESS and semi‐LASER in a healthy mouse using hyperpolarized [1‐^13^C]pyruvate. Repeat experiment to animal shown in Figure 5. Ten voxels (first seven shown, subsequent voxels had no signal due to overlap with previously excited/refocused planes) were placed in different organs/regions (A/B: T_2_w anatomical references, coronal and axial, with voxel locations overlayed). C/D: semi‐LASER/PRESS spectra. Spectra are shown line‐broadened (5 Hz) and normalized to background noise. E: 1° full‐volume excitation 3/6 s after start of injection. F: 90° full‐volume excitations after MRS showing that MV‐PRESS destroyed ca. 1.5 times more hyperpolarized magnetization than MV‐semi‐LASER.
**Table S6:** SNR values for comparison of multi‐voxel PRESS and semi‐LASER in a healthy mouse using hyperpolarized [1‐^13^C]pyruvate. Values for Figure S10. Voxels without SNR above noise level are not shown.
**Table S7:** FWHM values for comparison of multi‐voxel PRESS and semi‐LASER in a healthy mouse using hyperpolarized [1‐^13^C]pyruvate. Values for spectra shown in Figure S10 for voxels with an SNR above noise background threshold in both sequences as shown in Table S6.
**Figure S11:** Comparing multi‐voxel PRESS and semi‐LASER in a healthy mouse using hyperpolarized [1‐^13^C]pyruvate. Repeat experiment to animal shown in Figure 5. Ten voxels (first 7 shown, subsequent voxels were without signal due to overlap and destroyed magnetization) were placed in different organs/regions (A/B: T_2_w anatomical references, coronal and axial, with voxel locations overlayed). C/D: semi‐LASER/PRESS spectra. Spectra are shown line‐broadened (5 Hz) and normalized to background noise. E: 1° full‐volume excitation 5/11 s after start of injection. F: 90° full‐volume excitations after MRS showing that MV‐PRESS destroyed ca. 2 times more hyperpolarized magnetization than MV‐semi‐LASER.
**Table S8:** SNR values for comparison of multi‐voxel PRESS and semi‐LASER in a healthy mouse using hyperpolarized [1‐^13^C]pyruvate. Values for Figure S11. Voxels without SNR above noise level are not shown.
**Table S9:** FWHM values for comparison of multi‐voxel PRESS and semi‐LASER in a healthy mouse using hyperpolarized [1‐^13^C]pyruvate. Values for spectra shown in Figure S11 for voxels with an SNR above noise background threshold in both sequences as shown in Table S8.
**Table S10:** In vivo pH and SNR values obtained using MV‐semi‐LASER and [1,5‐^13^C_2_]Z‐OMPD in a healthy mouse shown in Figure 7. Uncertainties are obtained from fit accuracy.
**Figure S12:** Dynamic multi‐voxel PRESS and semi‐LASER in healthy mouse kidneys using hyperpolarized [1‐^13^C]pyruvate. A, B: Anatomical T_2_w reference images of both kidneys and voxel placement. C/D: semi‐LASER spectra from left and right kidney, respectively. E/F: PRESS spectra from left and right kidney. G/H: Peak area timecurves from spectral fits of pyruvate and lactate. Effective T_1_ computed for pyruvate peak intensities is increased for semi‐LASER spectra (6/4 s) versus PRESS spectra (3 s). Spectra are shown line‐broadened and normalized to the maximum intensity in semi‐LASER spectra.
