# Supplementary material for: 3D virtual biopsy of in vivo pH and metabolism using PRESS and semi‐LASER MRS of hyperpolarized 13C nuclei
Source: Magn Reson Med. 2025 May 1;94(3):880–94. doi: 10.1002/mrm.30544 (PMC12202722; doi:10.1002/mrm.30544)
Supplement: Supplementary file 1 — Table S1: PDAC MV‐PRESS mouse scan parameter overview. Coil 1 refers to 31 mm 1H/13C volume coil and coil 2 to 72 mm 1H/13C volume coil with 13C receiver array. Figure S1: Sequence diagrams of single‐voxel PRESS, multi‐voxel PRESS and multi‐voxel semi‐LASER with crusher gradients for PRESS sequences. Figure S2: Semi‐LASER crusher strength and adiabatic full passage RF‐pulse characteristics. In A‐G, magnitude spectra from a semi‐LASER voxel in an oil phantom are shown for varying crusher strengths in percent of the maximal gradient strength (590 mT/m). When compared to a PRESS spectrum (H), spurious echoes around 7–8 ppm at low crusher strengths are apparent (arrows in A–D). With increasing crusher strength, these echoes disappear but wave‐like artifacts around 3 and 7 ppm appear (arrows E‐G). Around 30%–40% of crusher strength both artifacts and spurious echoes are minimized. In I and J, the power for an AFP pulse was increased in an inversion recovery experiment to see where the pulse reaches inversion. For the proton channel (I), this happens at around 10 W (0.04 mT), while for carbon, 25 W (0.21 mT) is needed (J). K and L show the amplitude and phase of the 3.4 ms AFP HsN pulse used in this work. Figure S3: Multi‐voxel MRS planning tool overview. Anatomical reference images can be loaded and voxel position as well as slice locations overlaid for accurate overlay monitoring. Figure S4: 2D FID‐CSI point spread function and bleeding artifact simulation for a square shape. (A) K‐space encoding scheme for 2D FID‐CSI: each vertical column, as shown, of k‐space points is acquired in an up‐down alternating center‐out pattern, and then each horizontal row is acquired in a similar ordering left–right, giving substantially larger differences in remaining signal between k‐space points adjacent horizontally than vertically (B) Sample image of a uniform intensity square shape used for simulation of spatial bleeding and point spread function. (D) Convolution of point spread fun [file MRM-94-880-s001.docx]

# Supplemental Information

| Animal/Day | Coil | Sequence started (seconds after injection start) | Voxel size [mm^3^] | Figure |
| --- | --- | --- | --- | --- |
| Pyruvate Animal 1/1 | 1 | 29 | 2x2x2 | S6/7 |
| Pyruvate Animal 1/2 | 1 | 15 | 2x2x2 | S6/7 |
| Pyruvate Animal 2 | 2 | 22 | 2x2x2 | S6/7 |
| Pyruvate Animal 3/1 | 2 | 22 | 2x2x2 | S6/7 |
| Pyruvate Animal 3/2 | 2 | 22 | 2x2x2 | S6/7 |
| Pyruvate Animal 4 | 2 | 22 | 2x2x2 | S6/7 |
| Pyruvate Animal 5 | 1 | 21 | 2x2x2 | S6/7 |
| Pyruvate Animal 6 | 1 | 21 | 2x2x2 | S6/7 |
| OMPD Animal 1 | 1 | 17 | 2.5x2.5x2.5 | 4 |
| OMPD Animal 2 | 1 | 19 | 2x2x2 | S5 |

**Table S1: PDAC MV-PRESS mouse scan parameter overview.** Coil 1 refers to 31 mm ^1^H/^13^C volume coil and coil 2 to 72 mm ^1^H/^13^C volume coil with ^13^C receiver array.


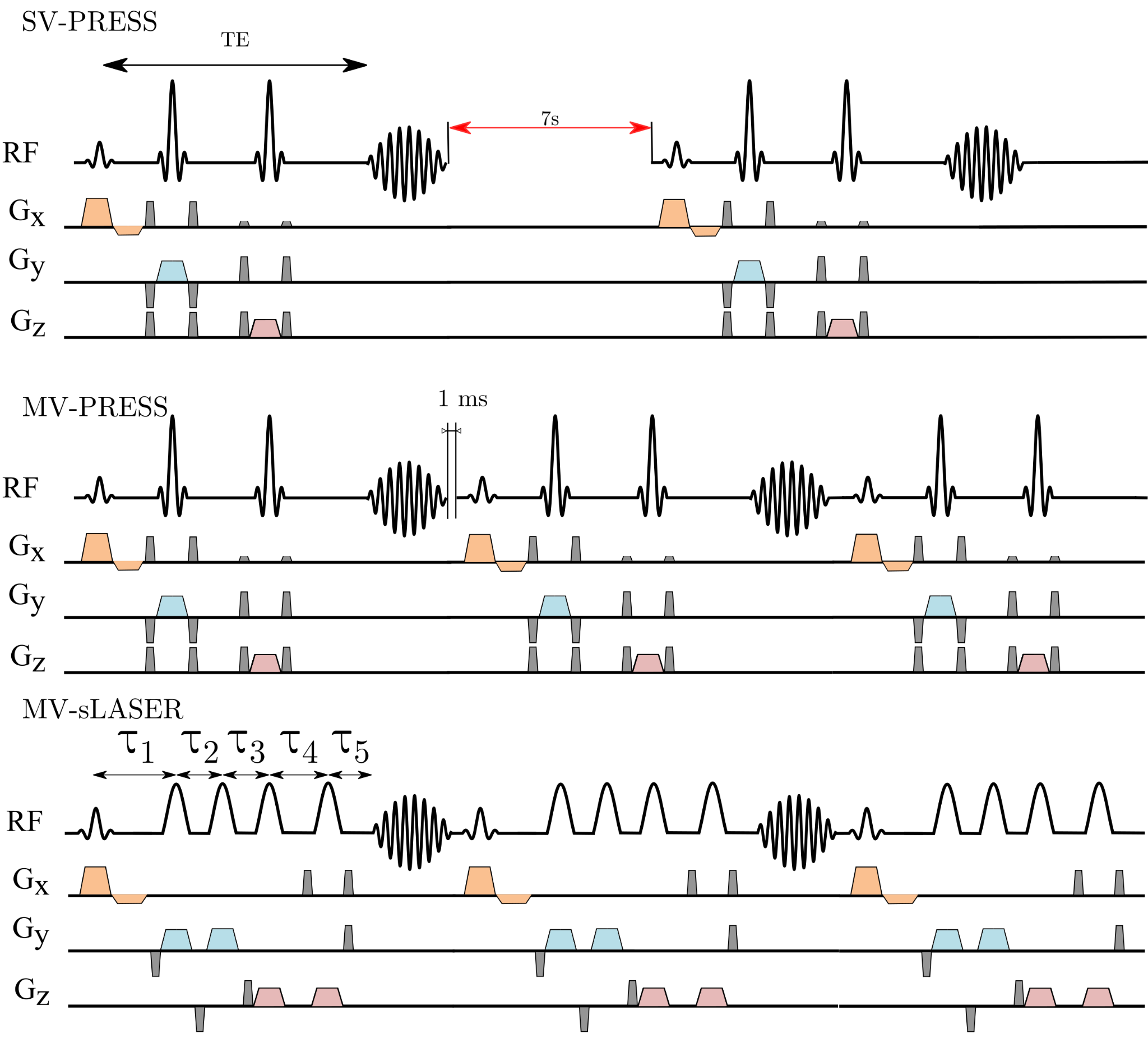


**Figure S1: Sequence diagrams of single-voxel PRESS, multi-voxel PRESS and multi-voxel semi-LASER with crusher gradients for PRESS sequences.**


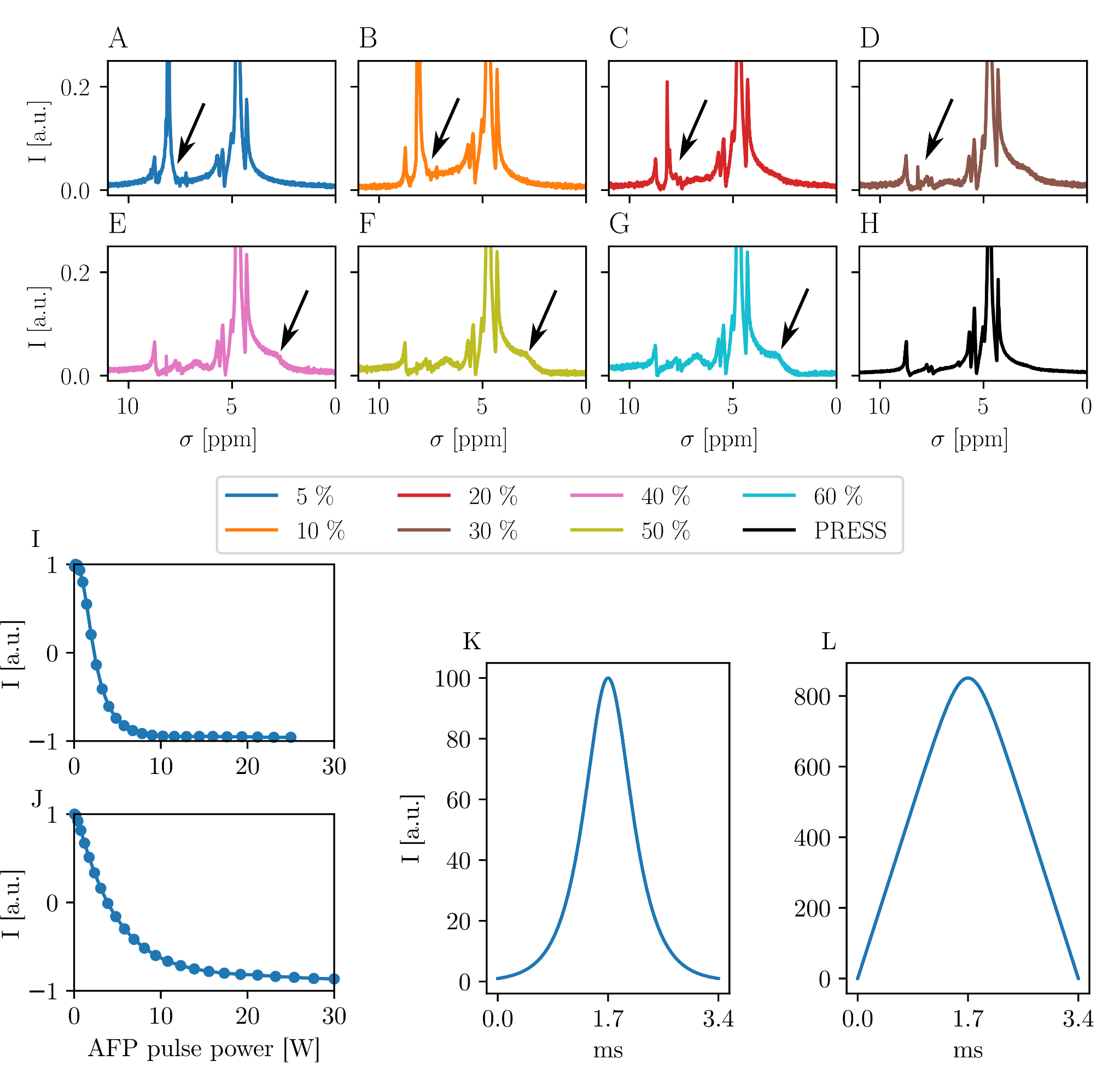


**Figure S2: semi-LASER crusher strength and adiabatic full passage RF-pulse characteristics.** In A-G, magnitude spectra from a semi-LASER voxel in an oil phantom are shown for varying crusher strengths in percent of the maximal gradient strength (590 mT/m). When compared to a PRESS spectrum (H), spurious echoes around 7-8 ppm at low crusher strengths are apparent (arrows in A-D). With increasing crusher strength, these echoes disappear but wave-like artifacts around 3 and 7 ppm appear (arrows E-G). Around 30-40 % of crusher strength both artifacts and spurious echoes are minimized. In I and J, the power for an AFP pulse was increased in an inversion recovery experiment to see where the pulse reaches inversion. For the proton channel (I), this happens at around 10 W (0.04 mT), while for carbon, 25 W (0.21 mT) is needed (J). K and L show the amplitude and phase of the 3.4 ms AFP HsN pulse used in this work.


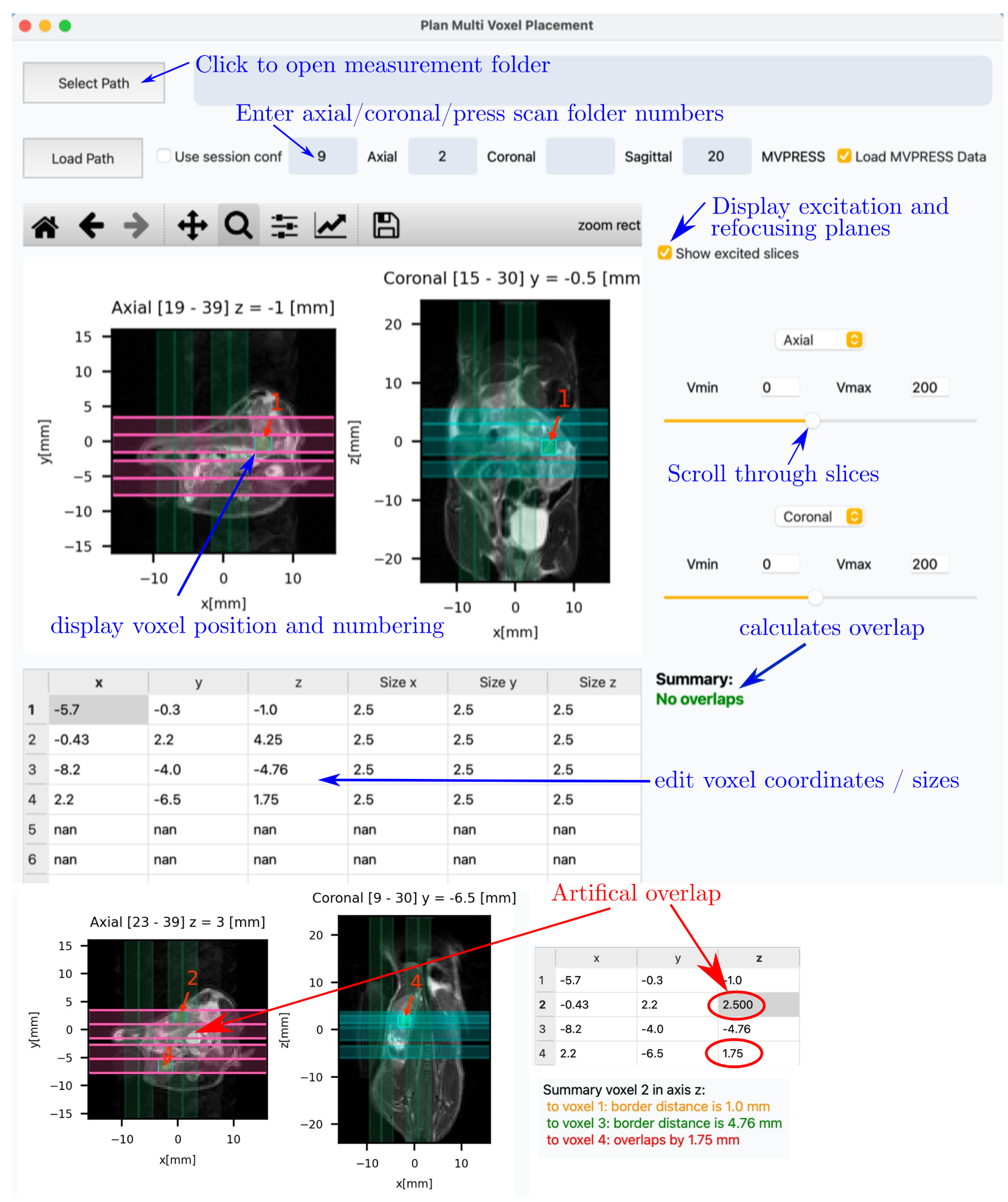


**Figure S3: Multi-voxel MRS planning tool overview.** Anatomical reference images can be loaded and voxel position as well as slice locations overlaid for accurate overlay monitoring.


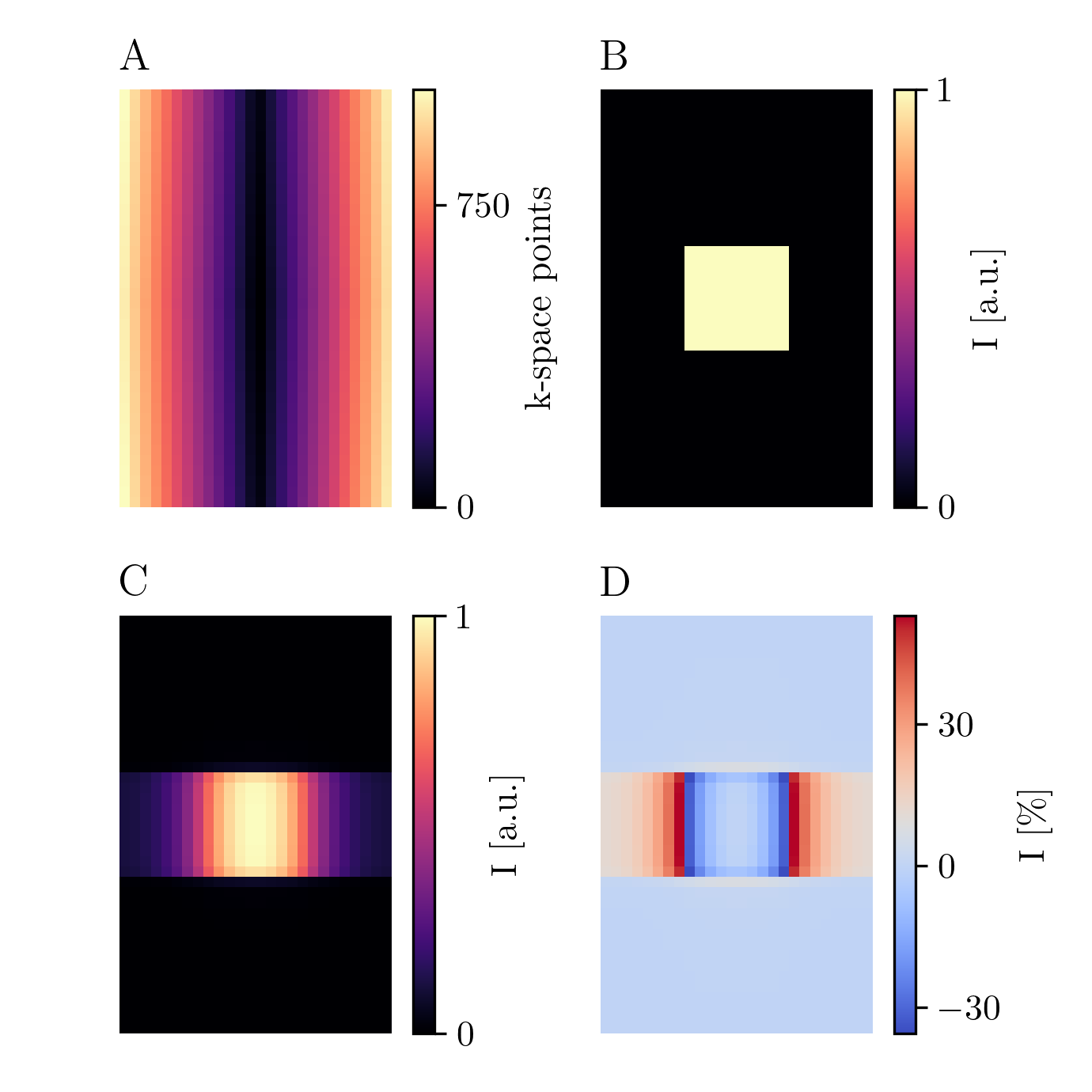


**Figure S4: 2D FID-CSI point spread function and bleeding artifact simulation for a square shape.** A: K-space encoding scheme for 2D FID-CSI: each vertical column, as shown, of k-space points is acquired in a up-down alternating centre-out pattern, and then each horizontal row is acquired in a similar ordering left-right, giving substantially larger differences in remaining signal between k-space points adjacent horizontally than vertically B: Sample image of a uniform intensity square shape used for simulation of spatial bleeding and point spread function. D: Convolution of point spread function and test image, with signal spatial bleeding visible in left-right direction, giving effective spatial resolution worse than the nominal voxel size and spacing, also highlighted in the subtraction of images B and C shown in D.


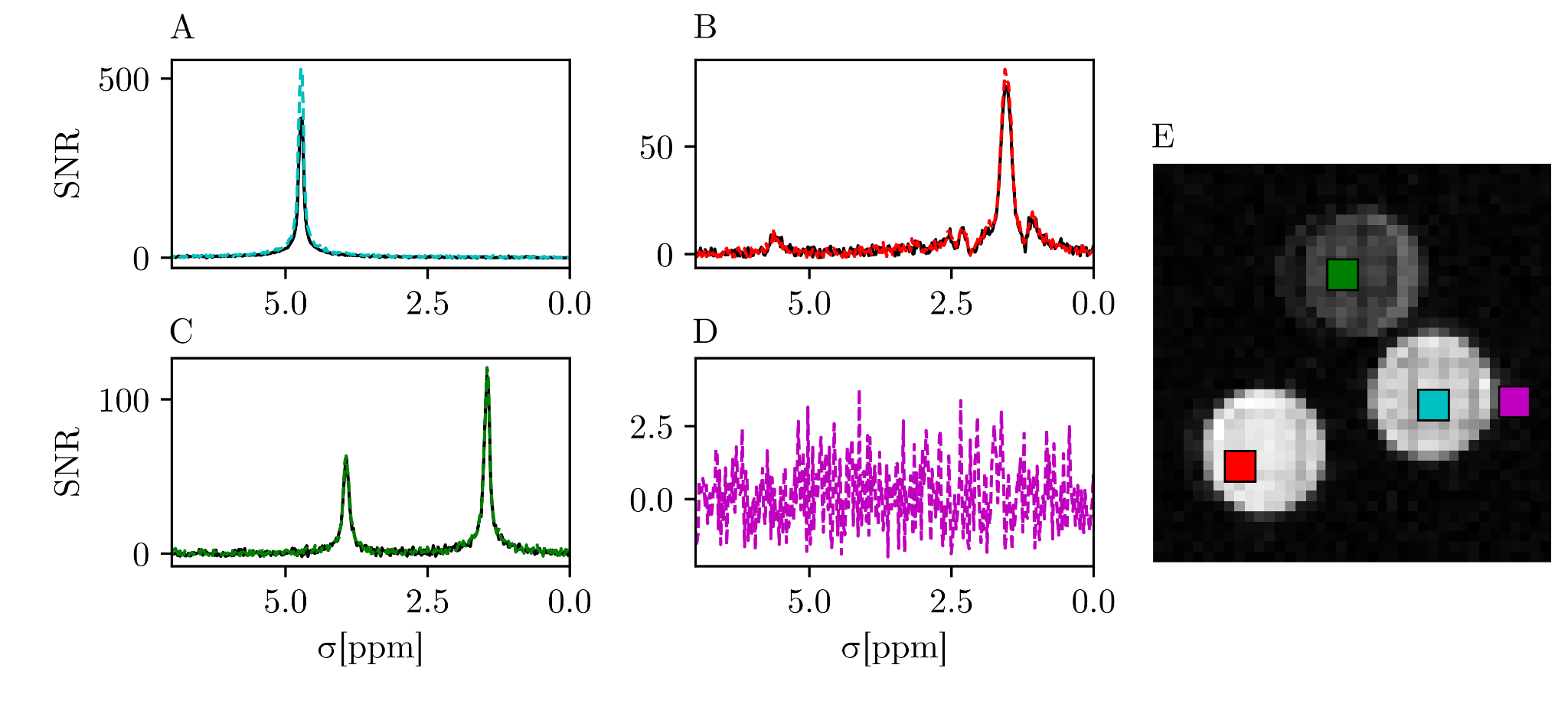


**Figure S5: Validation of multi-voxel PRESS against stock single-voxel PRESS sequence in a three-tube phantom.** Spectra at four locations (A: water, B: oil, C: ethanol, D: outside reference) were acquired using a single-voxel PRESS sequence (black lines) as well a a four-voxel MV-PRESS (colored lines) sequence at the same locations (T_1_w proton reference image shown in E). Spectra are virtually identical, as expected.


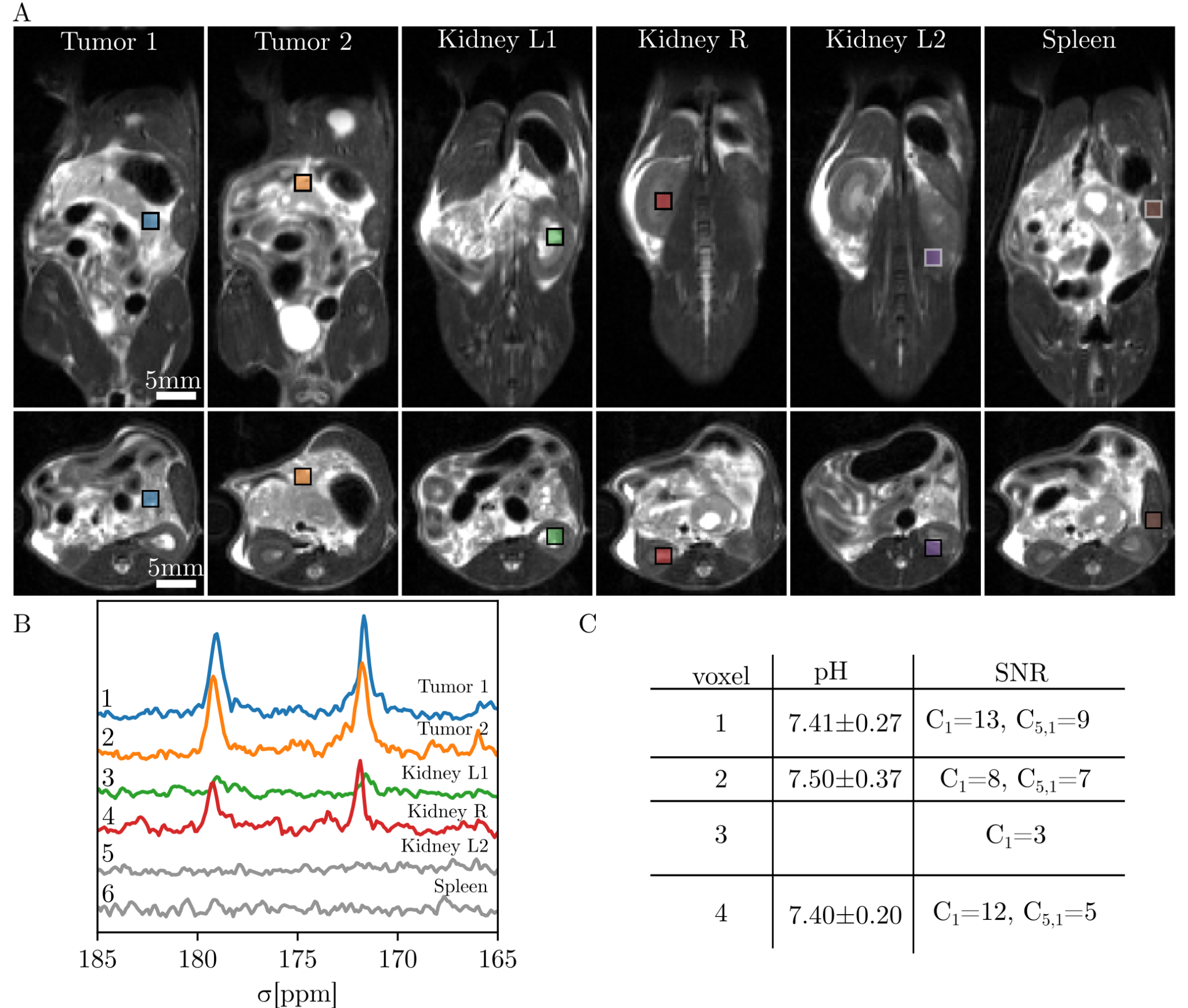


**Figure S6: In vivo pH measurement of PDAC animal 2 using Z-OMPD and MV-PRESS.** In A, the six voxel ROIs are shown. Note that the third voxel (Kidney L) is within an unusually bright area in the kidney, possibly due to kidney blockage related to the PDAC. No signal is recorded in this ROI, in contrast to the other kidney (see spectra in B). C: pH values and SNR of OMPD peaks from multi-compartment fit.


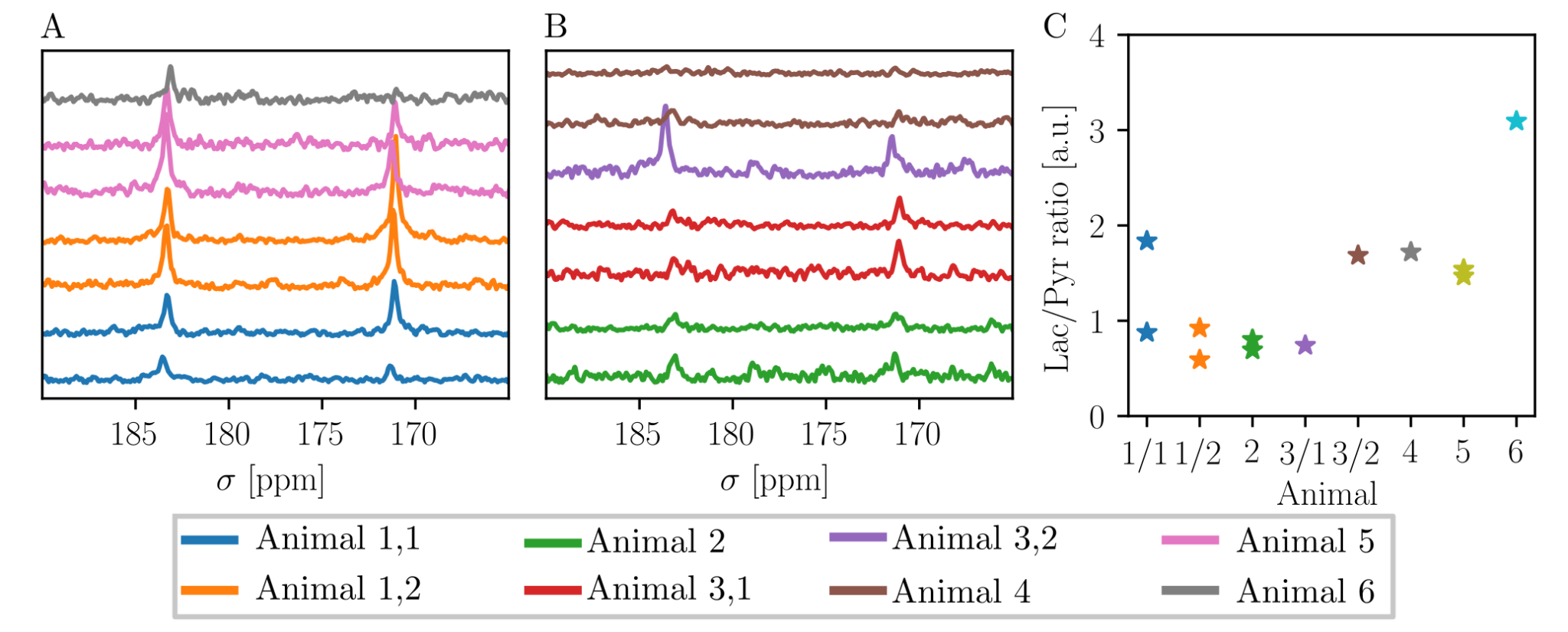


**Figure S7: Static detection of [1-^13^C]pyruvate and metabolites in PDAC mice using MV-PRESS.**Spectra acquired using MV-PRESS for two setups used (A: 31 mm ^1^H/^13^C volume coil, B: 72 mm ^1^H/^13^C volume coil with ^13^C receiver array, see Table S1). Lactate to pyruvate ratios for tumor lesions shown in C. Anatomical references with voxel positions of respective animals are shown in Figure S7. Reference voxel data acquired in kidneys, muscle tissue and other organs not shown here.


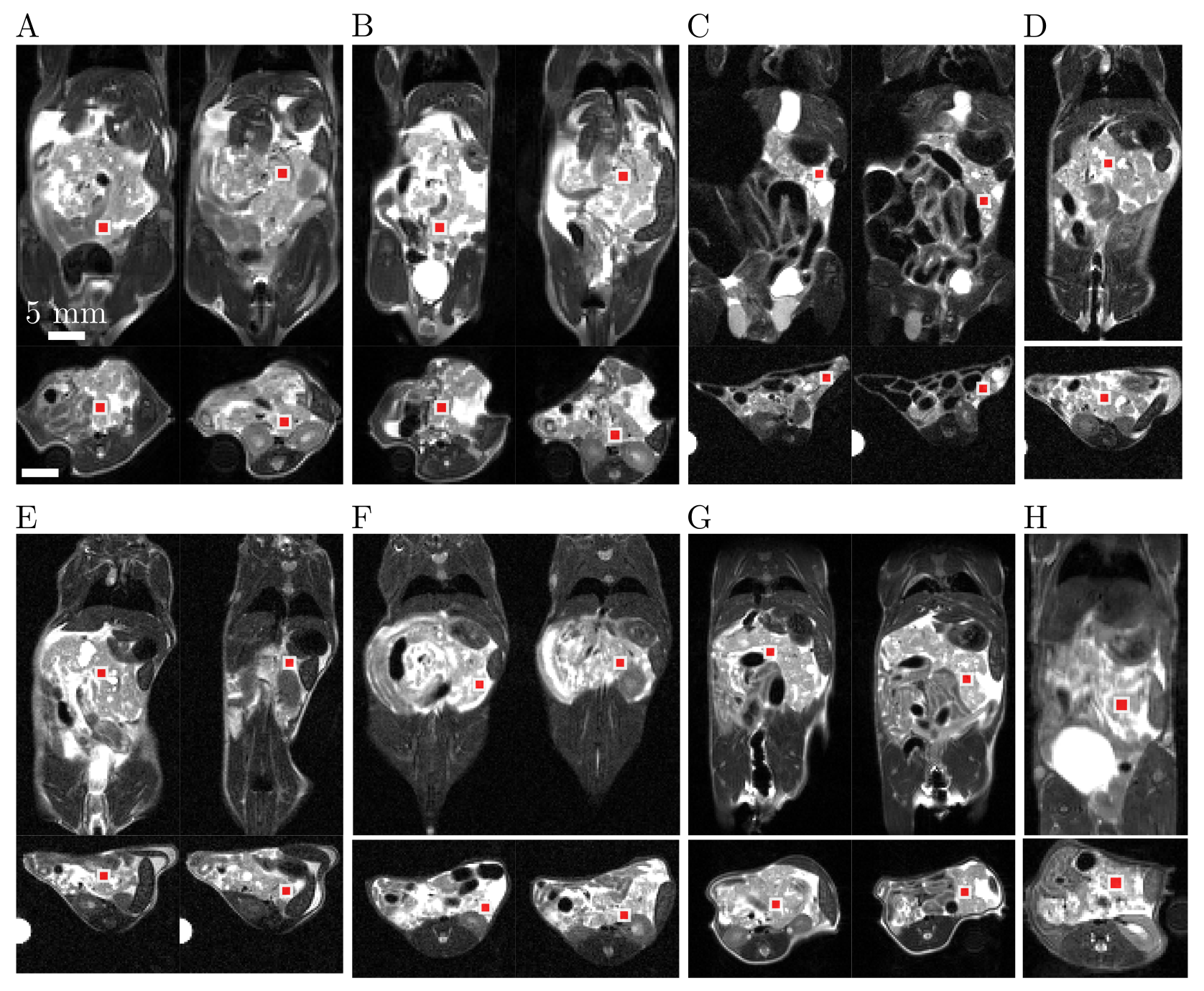


**Figure S8: Static detection of [1-^13^C]pyruvate and metabolites in PDAC mice using MV-PRESS.**Anatomical references for tumor voxel spectra shown in Figure S6. A: Animal 1,1. B: Animal 1,2. C: Animal 2. D: Animal 3,2. E: Animal 3,1. F: Animal 4. G: Animal 5. H: Animal 6.


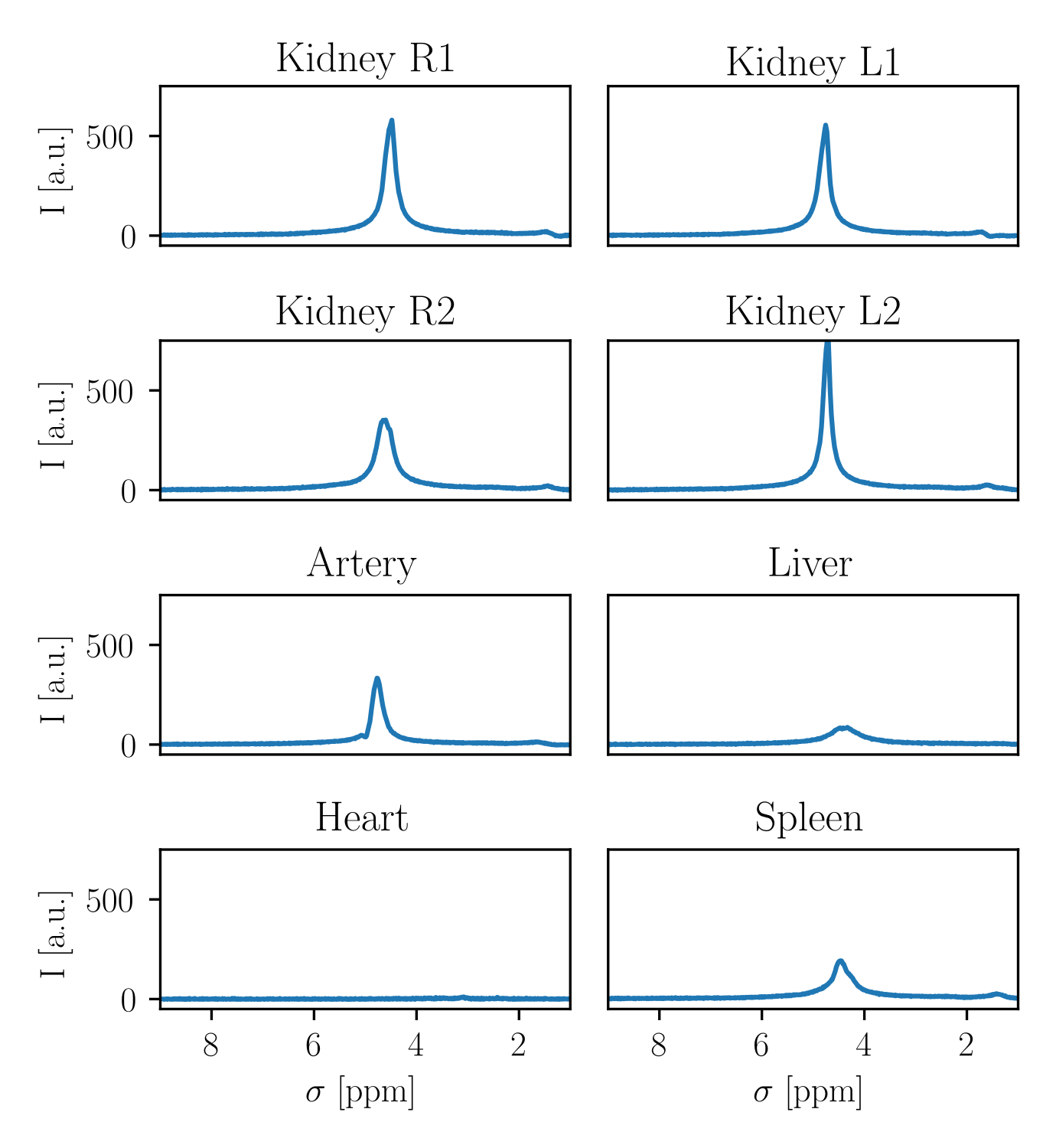


**Figure S9: Proton reference spectra at voxel locations of the animal shown in Figure 7.** Full width at half maximum (FWHM) values for the eight voxels range from 58-177 Hz at 7 T (83, 76, 118, 58, 69, 177, None, and 127) for (Kidney L1, Kidney R1, Kidney L2, Kidney R2, Artery, Liver, Heart and Spleen). Proton spectrum from the heart voxel is too low to compute a FWHM.

| **voxel** | **pH** | **SNR** |
| --- | --- | --- |
| 1, tumor 1 | 7.25±0.05, 7.03±0.04 | C_1_=38, C_5,1_=16, C_5,2_=9 |
| 2, tumor 2 | 7.15±0.58 | C_1_=14, C_5,1_=8 |
| 3, kidney right | 7.26±0.07, 6.99±0.05 | C_1_=35, C_5,1_=9, C_5,2_=11 |
| 4, kidney left | 7.26±0.11, 7.04±0.08 | C_1_=25,C_5,1_=5,C_5,2_=7 |
| 5, muscle |  | C_1_=4 |
| 6, bloodvessel |  | C_1_=10 |

**Table S2: In vivo pH values obtained using MV-PRESS and [1,5-^13^C_2_]Z-OMPD for PDAC mouse shown in Figure 4.** Uncertainties are obtained from fit accuracy.

|  | **SNR (semi-LASER/PRESS)** | | | |
| --- | --- | --- | --- | --- |
| **Location** | **Pyruvate** | **Lactate** | **Pyr-Hydrate** | **Alanine** |
| **Kidney R1** | 77 / 33 | 22 / 25 | 4 / 3 | / 4 |
| **Kidney L1** | 18 / 31 | 12 / 17 |  |  |
| **Kidney R2** | 12 / 3 | 4 / |  |  |
| **Kidney L2** | 25 / | 8 / |  |  |
| **Back muscle** |  |  |  |  |
| **Bloodvessel** | 9 / 10 |  |  |  |
| **Liver** |  |  |  | 3 / |

**Table S3: SNR values for comparison of multi-voxel PRESS and semi-LASER in a healthy mouse using hyperpolarized [1-^13^C]pyruvate**. Values for Figure 5.

|  |  | **Lactate-to-pyruvate ratio** | |
| --- | --- | --- | --- |
| **Animal** |  | **semi-LASER** | **PRESS** |
| **4, Figure 5** | **Kidney R1** | 0.4 | 1.1 |
|  | **Kidney L1** | 1.6 | 0.7 |
|  | **Kidney R2** | 0.5 |  |
|  | **Bloodvessel** | 0.5 |  |
| **12, Figure S10** | **Kidney L1** | 0.6 | 0.4 |
|  | **Kidney R1** | 1.5 | 0.4 |
|  | **Kidney R2** | 0.7 | 0.8 |
| **5, Figure S11** | **Kidney L1** | 0.5 | 0.8 |
|  | **Kidney R1** | 1.3 | 0.8 |
|  | **Kidney R2** | 1.2 | 0.5 |

**Table S4: Lactate to pyruvate ratios for comparison of multi-voxel PRESS and semi-LASER in a healthy mouse using hyperpolarized [1-^13^C]pyruvate**. Values for spectra shown in Figure 5, S10, S11 for voxels with an SNR above noise background threshold in both sequences.

|  | **FWHM [Hz at 7T] (semi-LASER/PRESS)** | | |
| --- | --- | --- | --- |
| **Location** | **Pyruvate** | **Lactate** | **Pyr-Hydrate** |
| **Kidney R1** | 20.3 / 22.5 | 27.0 / 32.9 | 27.9 / 24.9 |
| **Kidney L1** | 23.9 / 29.6 | 59.1 / 36.7 |  |
| **Kidney R2** | 31.0 / 29.3 |  |  |
| **Bloodvessel** | 19.9 / 15.5 |  |  |

**Table S5: FWHM values for comparison of multi-voxel PRESS and semi-LASER in a healthy mouse using hyperpolarized [1-^13^C]pyruvate**. Values for spectra shown in Figure 5 for voxels with an SNR above noise background in both sequences as shown in Table S3.


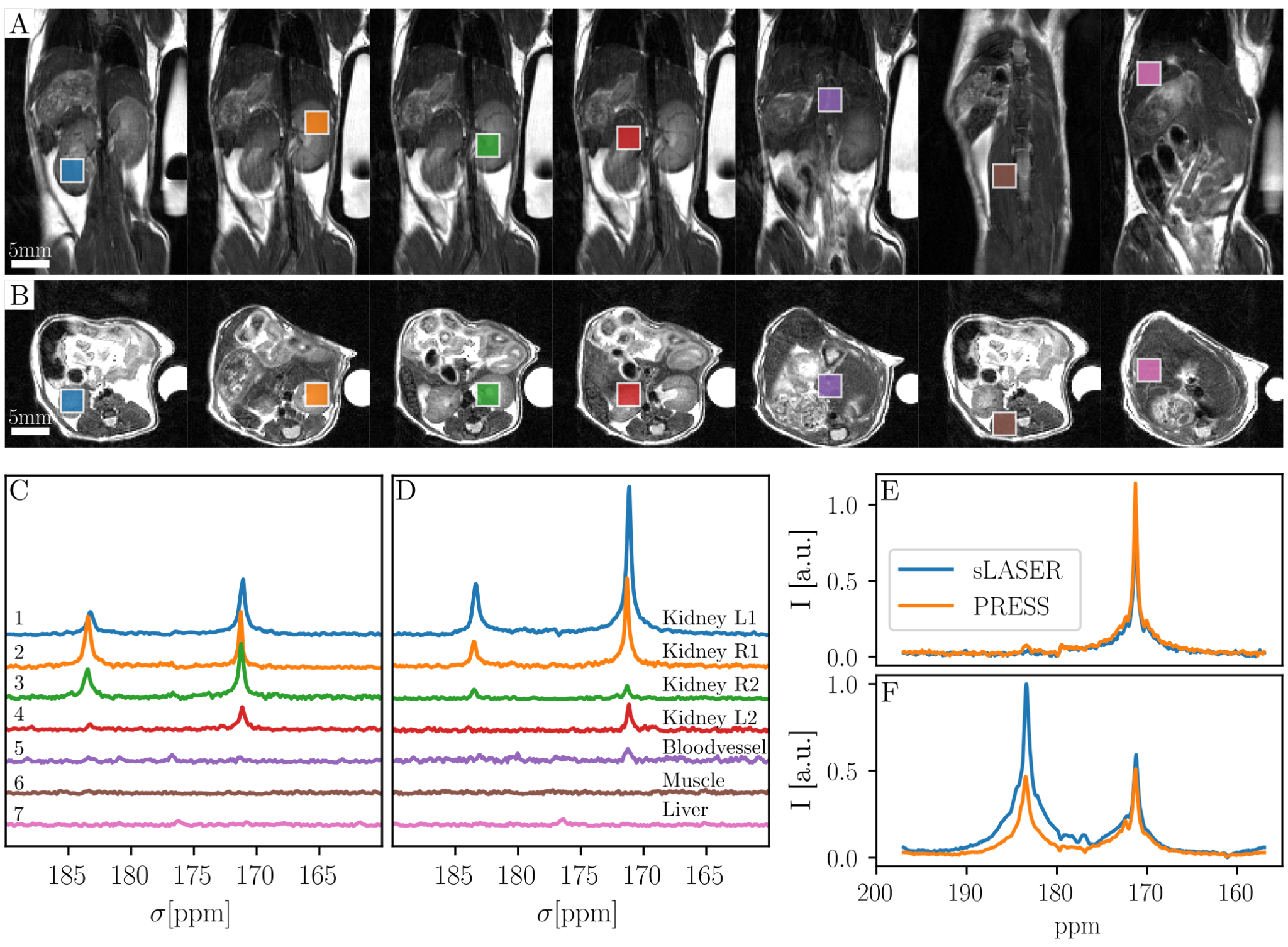


**Figure S10: Comparing multi-voxel PRESS and semi-LASER in a healthy mouse using hyperpolarized [1-^13^C]pyruvate. Repeat experiment to animal shown in Figure 5.** Ten voxels (first seven shown, subsequent voxels had no signal due to overlap with previously excited/refocused planes) were placed in different organs/regions (A/B: T_2_w anatomical references, coronal and axial, with voxel locations overlayed). C/D: semi-LASER/PRESS spectra. Spectra are shown line-broadened (5 Hz) and normalized to background noise. E: 1° full-volume excitation 3/6 s after start of injection. F: 90° full-volume excitations after MRS showing that MV-PRESS destroyed ca. 1.5 times more hyperpolarized magnetization than MV-semi-LASER.

|  | **SNR (semi-LASER/PRESS)** | | | |
| --- | --- | --- | --- | --- |
| **Location** | **Pyruvate** | **Lactate** | **Pyr-Hydrate** | **Alanine** |
| **Kidney L1** | 38 / 87 | 12 / 24 | / 5 |  |
| **Kidney R1** | 42 / 43 | 20 / 7 | 2 / | 2 / |
| **Kidney R2** | 19 / 8 | 7 / 4 |  |  |
| **Kidney L2** | 9 / 9 |  |  |  |

**Table S6: SNR values for comparison of multi-voxel PRESS and semi-LASER in a healthy mouse using hyperpolarized [1-^13^C]pyruvate**. Values for Figure S10. Voxels without SNR above noise level are not shown.

|  | **FWHM [Hz at 7T] (semi-LASER/PRESS)** | | |
| --- | --- | --- | --- |
| **Location** | **Pyruvate** | **Lactate** | **Pyr-Hydrate** |
| **Kidney L1** | 37 / 31 | 60 / 47 | / 36 |
| **Kidney R1** | 16 / 19 | 46 / 39 | 23 / |
| **Kidney R2** | 31 / 23 | 50 / 32 |  |
| **Kidney L2** | 34 / 20 |  |  |

**Table S7: FWHM values for comparison of multi-voxel PRESS and semi-LASER in a healthy mouse using hyperpolarized [1-^13^C]pyruvate**. Values for spectra shown in Figure S10 for voxels with an SNR above noise background threshold in both sequences as shown in Table S6.


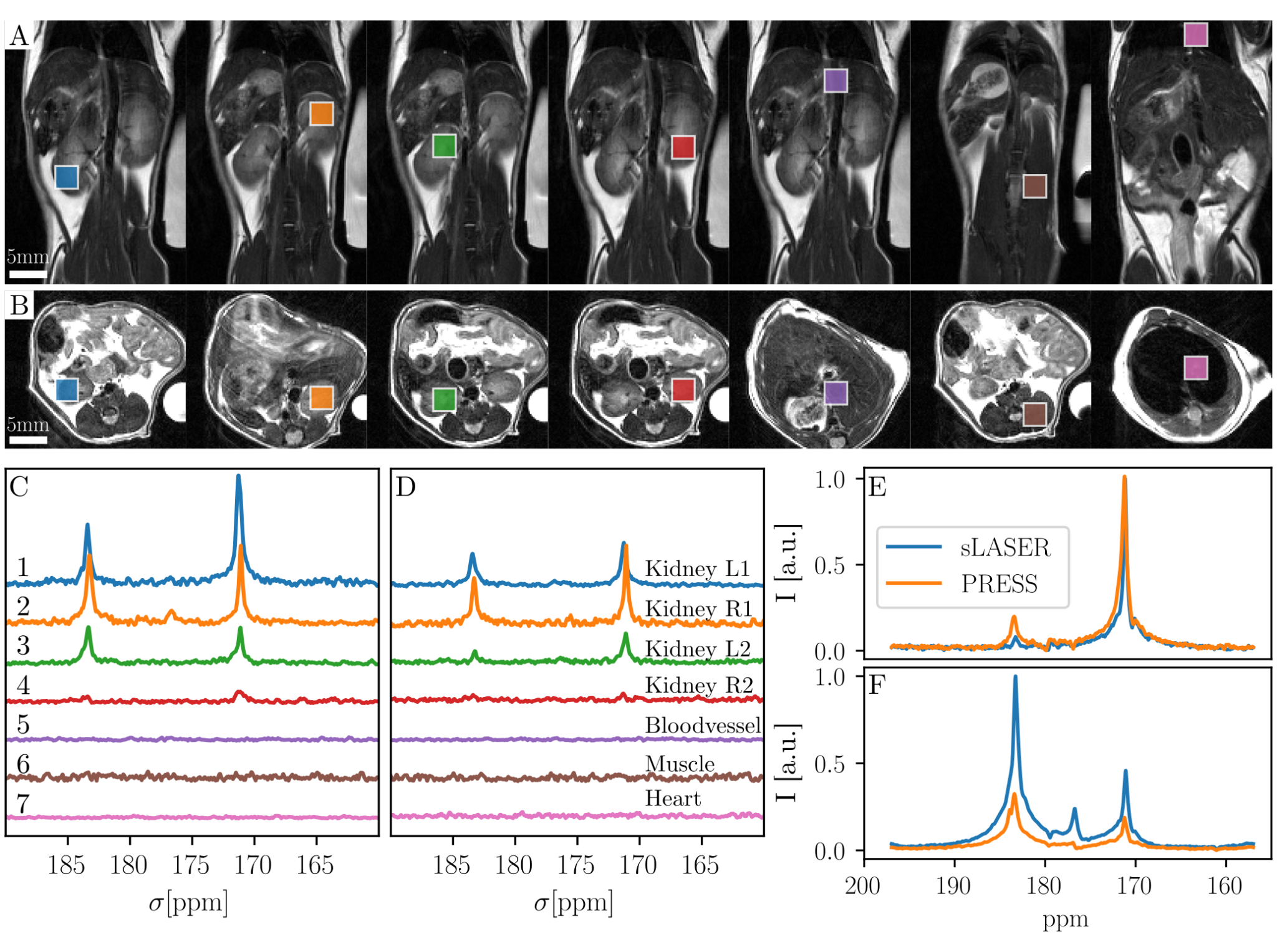
**Figure S11: Comparing multi-voxel PRESS and semi-LASER in a healthy mouse using hyperpolarized [1-^13^C]pyruvate. Repeat experiment to animal shown in Figure 5.** Ten voxels (first 7 shown, subsequent voxels were without signal due to overlap and destroyed magnetization). were placed in different organs/regions (A/B: T_2_w anatomical references, coronal and axial, with voxel locations overlayed). C/D: semi-LASER/PRESS spectra. Spectra are shown line-broadened (5 Hz) and normalized to background noise. E: 1° full-volume excitation 5/11 s after start of injection. F: 90° full-volume excitations after MRS showing that MV-PRESS destroyed ca. 2 times more hyperpolarized magnetization than MV-semi-LASER.

|  | **SNR (semi-LASER/PRESS)** | | | |
| --- | --- | --- | --- | --- |
| **Location** | **Pyruvate** | **Lactate** | **Pyr-Hydrate** | **Alanine** |
| **Kidney L1** | 19 / 16 | 11 / 10 |  |  |
| **Kidney R1** | 29 / 25 | 15 / 8 | 2 | 2 |
| **Kidney R2** | 12 / 8 | 10 / 2 |  |  |

**Table S8: SNR values for comparison of multi-voxel PRESS and semi-LASER in a healthy mouse using hyperpolarized [1-^13^C]pyruvate**. Values for Figure S11. Voxels without SNR above noise level are not shown.

|  | **FWHM [Hz at 7T] (semi-LASER/PRESS)** | | |
| --- | --- | --- | --- |
| **Location** | **Pyruvate** | **Lactate** | **Pyr-Hydrate** |
| **Kidney L1** | 41 / 33 | 30 / 41 |  |
| **Kidney R1** | 18 / 14 | 40 / 34 | / 35 |
| **Kidney R2** | 27 / 24 | 37 / 29 |  |

**Table S9: FWHM values for comparison of multi-voxel PRESS and semi-LASER in a healthy mouse using hyperpolarized [1-^13^C]pyruvate**. Values for spectra shown in Figure S11 for voxels with an SNR above noise background threshold in both sequences as shown in Table S8.

| **voxel** | **pH** | **SNR** |
| --- | --- | --- |
| 1, kidney right 1 | 7.32±0.02, 7.06±0.01, 6.58±0.01 | C_1_=41, C_5,1_=16, C_5,2_=12, C_5,3_=14 |
| 2, kidney left 1 | 7.42±0.08, 7.12±0.05 | C_1_=20, C_5,1_=15, C_5,2_=3 |
| 3, kidney right 2 | 7.09±0.08, 6.65±0.01, 6.51±0.01 | C_1_=27, C_5,1_=7, C_5,2_=30, C_5,3_=27 |
| 4, kidney left 2 | 7.35±0.05 | C_1_=21, C_5,1_=8 |
| 5, artery | 7.24±0.03, 6.81±0.02 | C_1_=34, C_5,1_=7, C_5,2_=3 |
| 6, liver |  | C_1_=3 |

**Table S10: In vivo pH and SNR values obtained using MV-semi-LASER and [1,5-^13^C_2_]Z-OMPD in a healthy mouse shown in Figure 7.** Uncertainties are obtained from fit accuracy.


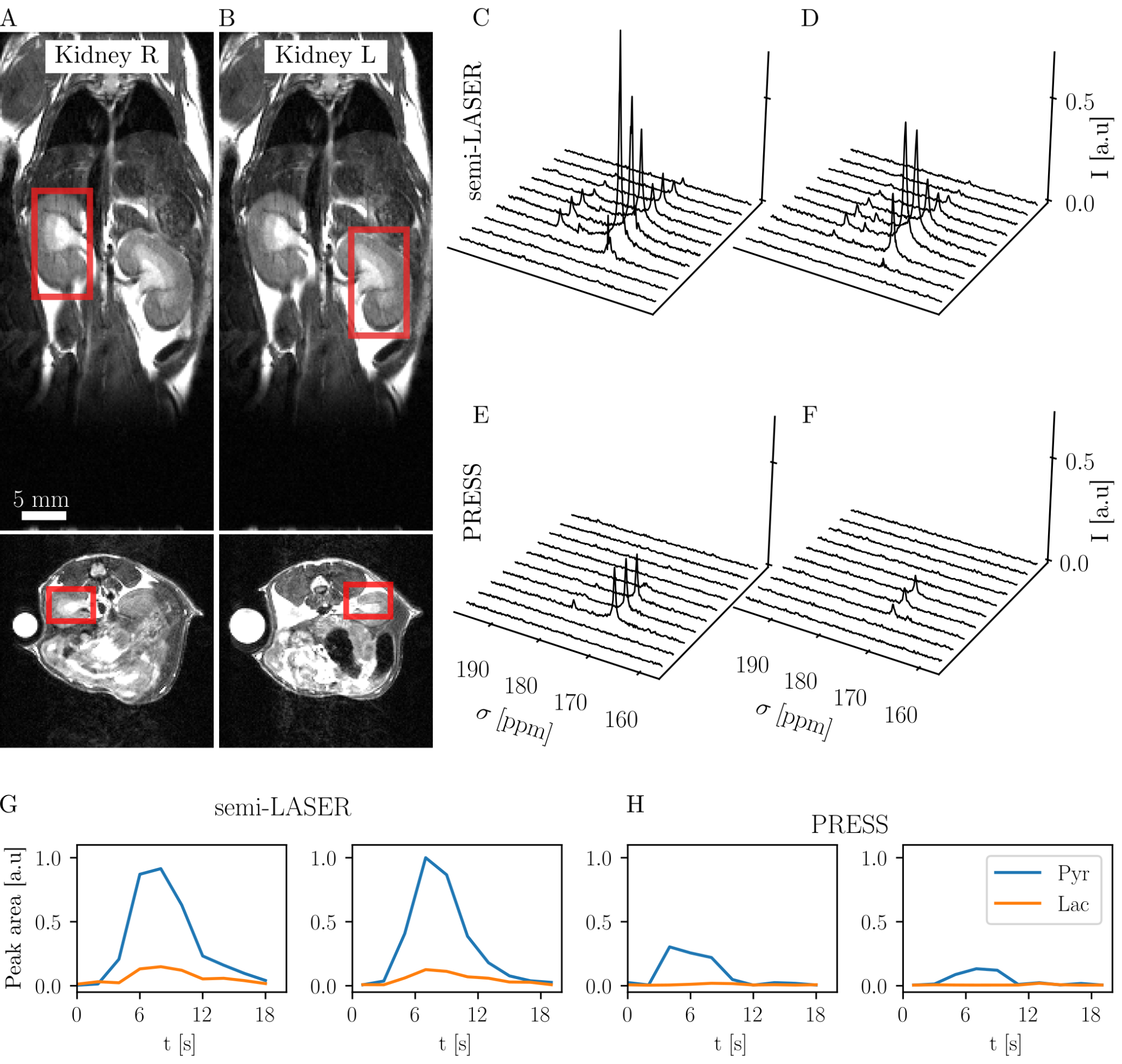


**Figure S12: Dynamic multi-voxel PRESS and semi-LASER in healthy mouse kidneys using hyperpolarized [1-^13^C]pyruvate.** A-B: Anatomical T_2_w reference images of both kidneys and voxel placement. C/D: semi-LASER spectra from left and right kidney, respectively. E/F: PRESS spectra from left and right kidney. G/H: Peak area timecurves from spectral fits of pyruvate and lactate. Effective T_1_ computed for pyruvate peak intensities is increased for semi-LASER spectra (6/4s) vs PRESS spectra (3s). Spectra are shown line-broadened and normalized to the maximum intensity in semi-LASER spectra.
